# Machine Learning in Amyotrophic Lateral Sclerosis: Achievements, Pitfalls, and Future Directions

**DOI:** 10.3389/fnins.2019.00135

**Published:** 2019-02-28

**Authors:** Vincent Grollemund, Pierre-François Pradat, Giorgia Querin, François Delbot, Gaétan Le Chat, Jean-François Pradat-Peyre, Peter Bede

**Affiliations:** ^1^Laboratoire d'Informatique de Paris 6, Sorbonne University, Paris, France; ^2^FRS Consulting, Paris, France; ^3^Laboratoire d'Imagerie Biomédicale, INSERM, CNRS, Sorbonne Université, Paris, France; ^4^APHP, Département de Neurologie, Hôpital Pitié-Salpêtrière, Centre Référent SLA, Paris, France; ^5^Northern Ireland Center for Stratified Medecine, Biomedical Sciences Research Institute Ulster University, C-TRIC, Altnagelvin Hospital, Londonderry, United Kingdom; ^6^Département de Mathématiques et Informatique, Paris Nanterre University, Nanterre, France; ^7^Modal'X, Paris Nanterre University, Nanterre, France; ^8^Computational Neuroimaging Group, Trinity College, Dublin, Ireland

**Keywords:** amyotrophic lateral sclerosis, machine learning, diagnosis, prognosis, risk stratification, clustering, motor neuron disease

## Abstract

**Background:** Amyotrophic Lateral Sclerosis (ALS) is a relentlessly progressive neurodegenerative condition with limited therapeutic options at present. Survival from symptom onset ranges from 3 to 5 years depending on genetic, demographic, and phenotypic factors. Despite tireless research efforts, the core etiology of the disease remains elusive and drug development efforts are confounded by the lack of accurate monitoring markers. Disease heterogeneity, late-stage recruitment into pharmaceutical trials, and inclusion of phenotypically admixed patient cohorts are some of the key barriers to successful clinical trials. Machine Learning (ML) models and large international data sets offer unprecedented opportunities to appraise candidate diagnostic, monitoring, and prognostic markers. Accurate patient stratification into well-defined prognostic categories is another aspiration of emerging classification and staging systems.

**Methods:** The objective of this paper is the comprehensive, systematic, and critical review of ML initiatives in ALS to date and their potential in research, clinical, and pharmacological applications. The focus of this review is to provide a dual, clinical-mathematical perspective on recent advances and future directions of the field. Another objective of the paper is the frank discussion of the pitfalls and drawbacks of specific models, highlighting the shortcomings of existing studies and to provide methodological recommendations for future study designs.

**Results:** Despite considerable sample size limitations, ML techniques have already been successfully applied to ALS data sets and a number of promising diagnosis models have been proposed. Prognostic models have been tested using core clinical variables, biological, and neuroimaging data. These models also offer patient stratification opportunities for future clinical trials. Despite the enormous potential of ML in ALS research, statistical assumptions are often violated, the choice of specific statistical models is seldom justified, and the constraints of ML models are rarely enunciated.

**Conclusions:** From a mathematical perspective, the main barrier to the development of validated diagnostic, prognostic, and monitoring indicators stem from limited sample sizes. The combination of multiple clinical, biofluid, and imaging biomarkers is likely to increase the accuracy of mathematical modeling and contribute to optimized clinical trial designs.

## 1. Introduction

Amyotrophic Lateral Sclerosis (ALS) is an adult-onset multi-system neurodegenerative condition with predominant motor system involvement. In Europe, its incidence varies between 2 or 3 cases per 100 000 individuals (Hardiman et al., 2017) and its prevalence is between 5 and 8 cases per 100 000 (Chiò et al., 2013b). An estimated 450 000 people are affected by ALS worldwide according to the ALS Therapy Development Institute. While no unifying pathogenesis has been described across the entire spectrum of ALS phenotypes, the incidence of the condition is projected to rise in the next couple of decades (Arthur et al., 2016) highlighting the urgency of drug development and translational research. Given the striking clinical and genetic heterogeneity of ALS, the considerable differences in disability profiles and progression rates, flexible individualized care strategies are required in multidisciplinary clinics (den Berg et al., 2005), and it is also possible that precision individualized pharmaceutical therapies will be required.

**Figure 1:**
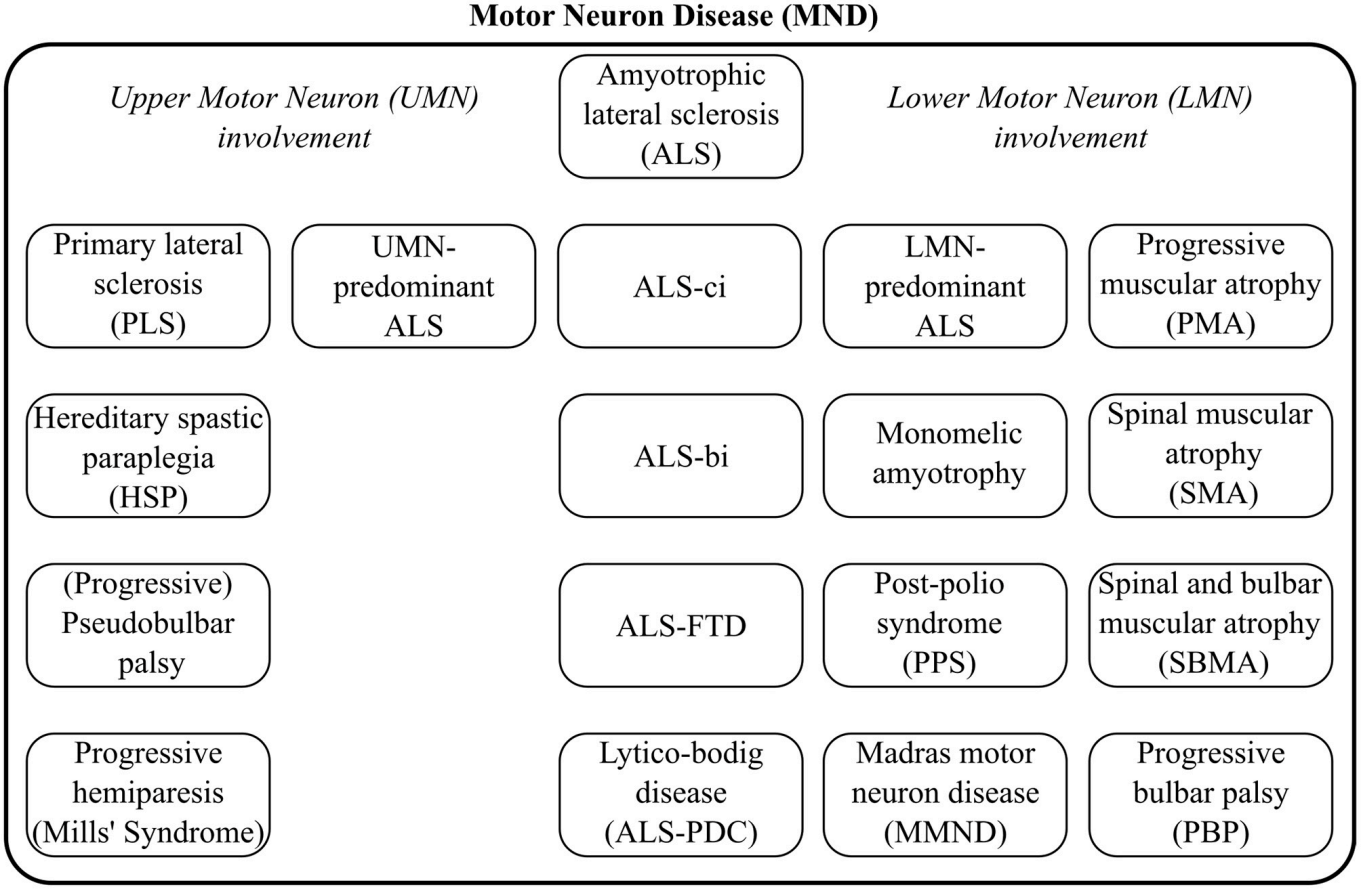
The clinical heterogeneity of Motor Neuron Disease common phenotypes and distinct syndromes.

Depending on geographical locations, the terms “ALS” and “Motor Neuron Disease” (MND) are sometimes used interchangeably, but MND is the broader label, encompassing a spectrum of conditions, as illustrated by Figure 1. The diagnosis of ALS requires the demonstration of Upper (UMN) and Lower Motor Neuron (LMN) dysfunction. The diagnostic process is often protracted. The careful consideration of potential mimics and ruling out alternative neoplastic, structural, and infective etiologies, is an important priority (Hardiman et al., 2017). ALS often manifests with subtle limb or bulbar symptoms and misdiagnoses and unnecessary interventions in the early stage of the disease are not uncommon (Zoccolella et al., 2006; Cellura et al., 2012). Given the limited disability in early-stage ALS, many patients face a long diagnostic journey from symptom onset to definite diagnosis which may otherwise represent a valuable therapeutic window for neuroprotective intervention. Irrespective of specific healthcare systems the average time interval from symptoms onset to definite diagnosis is approximately 1 year (Traynor et al., 2000). ALS is now recognized as a multi-dimensional spectrum disorder. From a cognitive, neuropsychological perspective, an ALS-Frontotemporal Dementia (FTD) spectrum exists due to shared genetic and pathological underpinnings. Another important dimension of the clinical heterogeneity of ALS is the proportion of UMN / LMN involvement which contributes to the spectrum of Primary Lateral Sclerosis (PLS), UMN-predominant ALS, classical ALS, LMN-predominant ALS, and Progressive Muscular Atrophy (PMA), as presented in Figure 1.

The genetic profile of MND patients provides another layer of heterogeneity. Specific genotypes such as those carrying the *C9orf72* hexanucleotide expansions or those with Super Oxide Dismutase 1 (*SOD1*) mutations have been associated with genotype-specific clinical profiles. These components of disease heterogeneity highlight the need for individualized management strategies and explain the considerable differences in prognostic profiles. Differences in survival due to demographic, phenotypic, and genotypic factors are particularly important in pharmaceutical trials so that the “treated” and “placebo-control” groups are matched in this regard.

With the ever increasing interest in Machine Learning (ML) models, a large number of research papers have been recently published using ML, classifiers, and predictive modeling in ALS (Bede, 2017). However, as these models are usually applied to small data sets by clinical teams, power calculations, statistical assumptions, and mathematical limitations are seldom discussed in sufficient detail. Accordingly our objective is the synthesis of recent advances, discussion of common shortcomings and outlining future directions. The overarching intention of this paper is to outline best practice recommendations for ML applications in ALS.

## 2. Methods

Machine learning is a rapidly evolving field of applied mathematics focusing on the development and implementation of computer software that can learn autonomously. Learning is typically based on training data sets and a set of specific instructions. In medicine, it has promising diagnostic, prognostic, and risk stratification applications and it has been particularly successful in medical oncology (Kourou et al., 2015).

### 2.1. Main Approaches

Machine learning encompasses two main approaches; “supervised” and “unsupervised” learning. The specific method should be carefully chosen based on the characteristics of the available data and the overall study objective.

**Figure 2:**
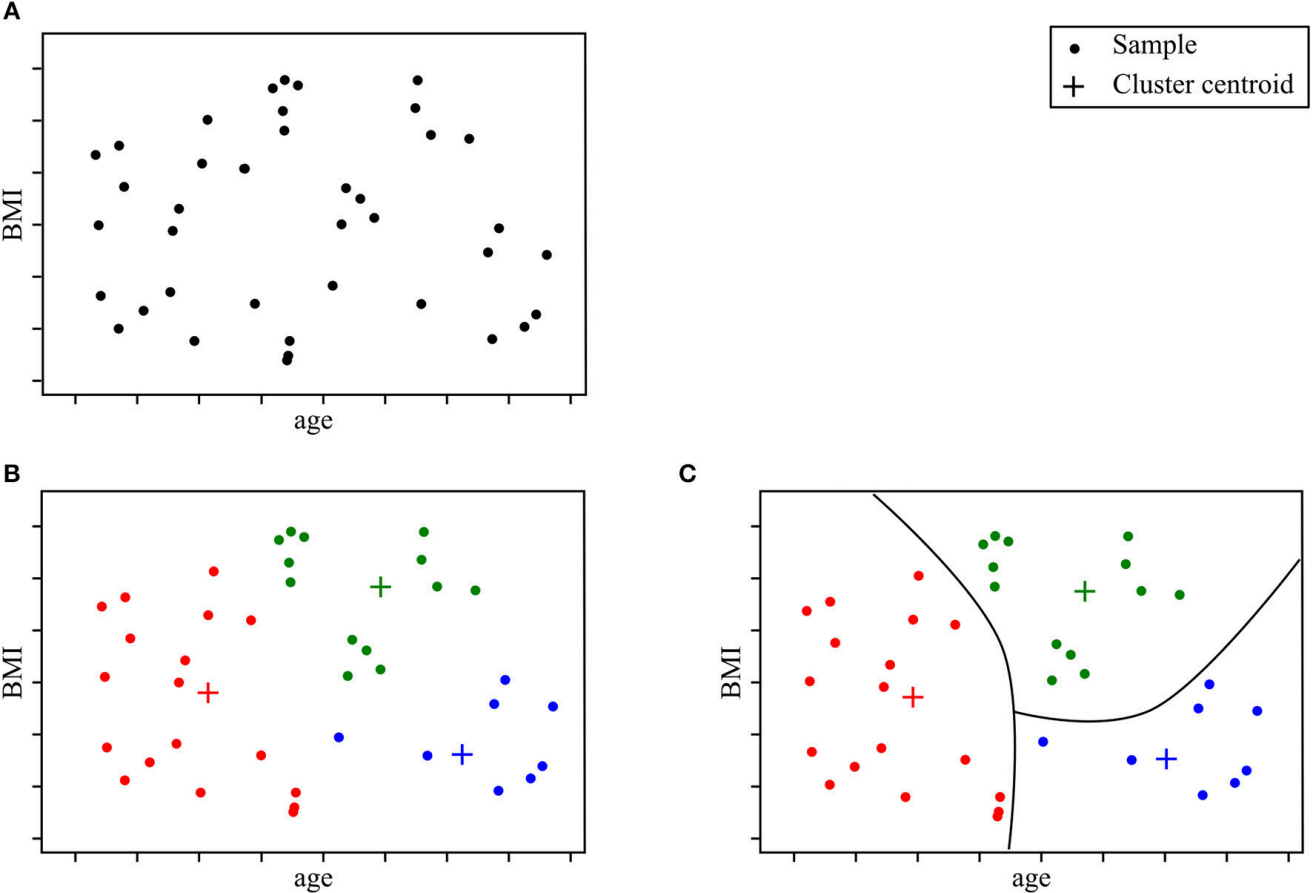
Clustering model for patient stratification. The available data consist of basic clinical features; age and BMI. Given this specific ALS patient population, the objective is to explore if patients segregate into specific subgroups. After running a clustering algorithm, we obtain clusters and cluster memberships for each patient. Further analysis of shared traits within the same cluster can help identify novel disease phenotypes. **(A)** Initial data samples without output. **(B)** Identify cluster and cluster membership. **(C)** Stratify samples based on shared feature traits.

“Unsupervised learning” aims to learn the structure of the data in the absence of either a well-defined output or feedback (Sammut and Webb, 2017). Unsupervised learning models can help uncover novel arrangements in the data which in turn can offer researchers new insights into the problem itself. Unsupervised learning can be particularly helpful in addressing patient stratification problems. Clustering methods can be superior to current clinical criteria, which are often based on a limited set of clinical observations, rigid thresholds, and conservative inclusion/exclusion criteria for class membership. The K-means algorithm is one of the most popular methods. It recursively repeats two steps until a stopping criterion is met. First, samples are assigned to the closest cluster, which are randomly initialized, then cluster centers are computed based on the centroid of samples belonging to each cluster. Unsupervised learning methods have been successfully used in other fields of medicine (Gomeni and Fava, 2013; Marin et al., 2015; Beaulieu-Jones and Greene, 2016; Ong et al., 2017; Westeneng et al., 2018). Figure 2 represents an example of a patient stratification scheme using an unsupervised learning algorithm.

Supervised learning focuses on mapping inputs with outputs using training data sets (Sammut and Webb, 2017). Supervised learning problems can be divided into either classification or regression problems. Classification approaches allocate test samples into specific categories or sort them in a meaningful way (Sammut and Webb, 2017). The possible outcomes of the modeled function are limited to a set of predefined categories. For example, in the context of ALS, a possible classification task is to link demographic variables, clinical observations, radiological measures, etc. to diagnostic labels such as “ALS,” “FTD,” or “healthy.” Schuster et al. (2016b), Bede et al. (2017), Ferraro et al. (2017), and Querin et al. (2018) have implemented diagnostic models to discriminate between patients with ALS and healthy subjects. Regression problems on the other hand, deal with inferring a real-valued function dependent on input variables, which can be dependent or independent of one another (Sammut and Webb, 2017). For instance, in the context of prognosis, a possible regression task could consist of designing a model which accurately predicts motor decline based on clinical observations (Hothorn and Jung, 2014; Taylor A. A. et al., 2016). When a regression task deals with time-related data sequences, often called “longitudinal data” in a medical context, it is referred to as “time series forecasting.” The core characteristics of the data, which are most likely to define group-membership are referred to as “features.”

### 2.2. Common Machine Learning Models

While a plethora of ML models have been developed and successfully implemented for economic, industrial, and biological applications (Hastie et al., 2009; Bishop, 2016; Goodfellow et al., 2017), this paper primarily focuses on ML methods utilized in ALS research. These include Random Forests (RF) (Hothorn and Jung, 2014; Ko et al., 2014; Beaulieu-Jones and Greene, 2016; Sarica et al., 2016; Taylor A. A. et al., 2016; Ferraro et al., 2017; Fratello et al., 2017; Huang et al., 2017; Jahandideh et al., 2017; Seibold et al., 2017; Pfohl et al., 2018; Querin et al., 2018), Support Vector Machines (SVM) (Srivastava et al., 2012; Welsh et al., 2013; Beaulieu-Jones and Greene, 2016; Bandini et al., 2018; D'hulst et al., 2018), Neural Networks (NN) (Beaulieu-Jones and Greene, 2016; van der Burgh et al., 2017), Gaussian Mixture Models (GMM) (Huang et al., 2017), Boosting methods (Jahandideh et al., 2017; Ong et al., 2017), k-Nearest Neighbors (k-NN) (Beaulieu-Jones and Greene, 2016; Bandini et al., 2018). Generalized linear regression models are also commonly used (Gordon et al., 2009; Taylor A. A. et al., 2016; Huang et al., 2017; Li et al., 2018; Pfohl et al., 2018), but will not be presented here. Please refer to Bishop (2016) for additional information on linear modeling. Our review of ML model families does not intend to be comprehensive with regards to ML models utilized in other medical subspecialties. Additional models with successful implementation in neurological conditions include Latent Factor models (Geifman et al., 2018) and Hidden Markov Models (HMM) (Martinez-Murcia et al., 2016) which have been successfully implemented in Alzheimer disease cohorts.

#### 2.2.1. Random Forests

**Figure 3:**
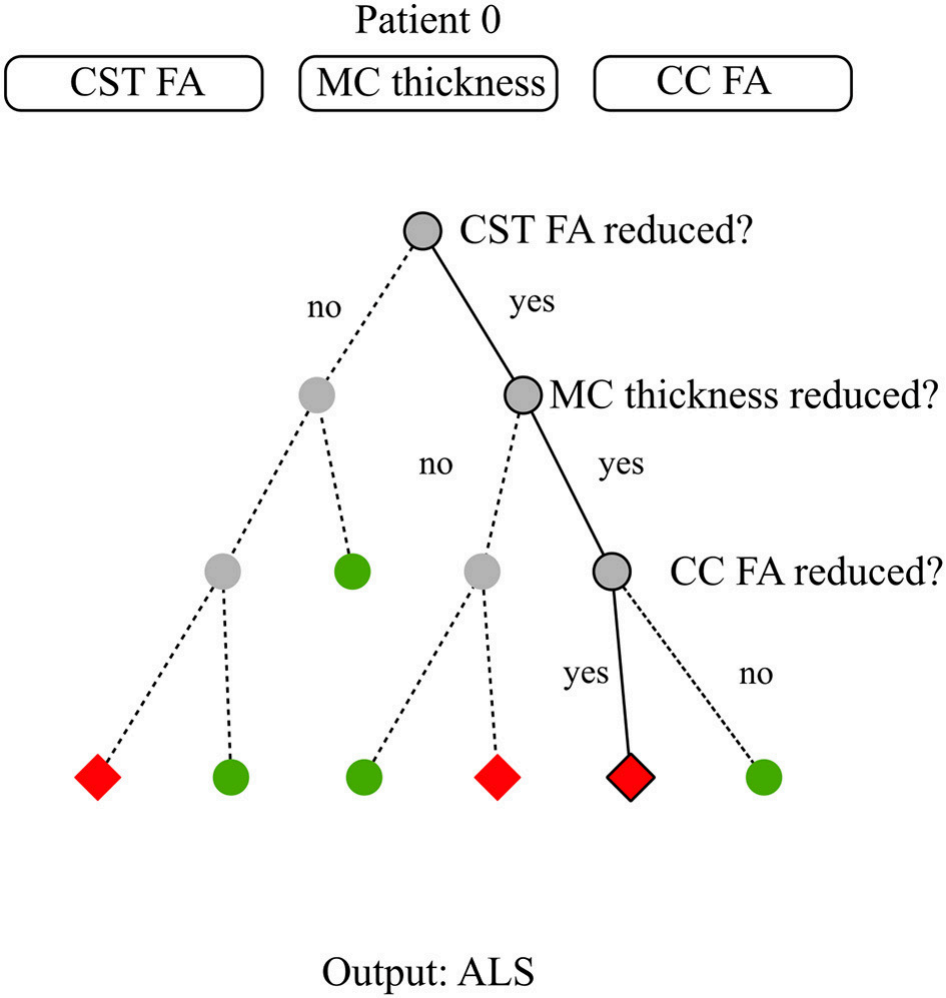
Decision tree model for diagnosis. The available data consist of three basic neuroimaging features: average Corticospinal Tract (CST) Fractional Anisotropy (FA), Motor Cortex (MC) thickness, and average Corpus Callosum (CC) FA. For patient 0, these features are reduced CST FA, reduced MC thickness, reduced CC FA. The target is to classify subjects between healthy and ALS subjects. Establishing a diagnosis requires to run through the decision tree till there are no more questions to answer. At step 1, the closed question directs to the right node due to patient 0's CST pathology. At step 2, the closed question directs to the right node due to patient 0's MC pathology. At step 3, the closed question directs to the left node due to patient 0 CC involvement. Step 3 is the last step as there is no more steps below. The diagnosis for patient 0 is the arrival cell value which is ALS.

Tree-based methods partition the input space into sets that minimize an error function, impurity, or entropy (Hastie et al., 2009). A decision tree is a tree-based method that can be described as a series of bifurcations with yes/no questions. To compute the output of a data sample, one needs to start at the top of the tree, and iteratively decide where to go next based on the answer. Figure 3 illustrates an example of a decision tree for diagnosis modeling in ALS.

**Figure 4:**
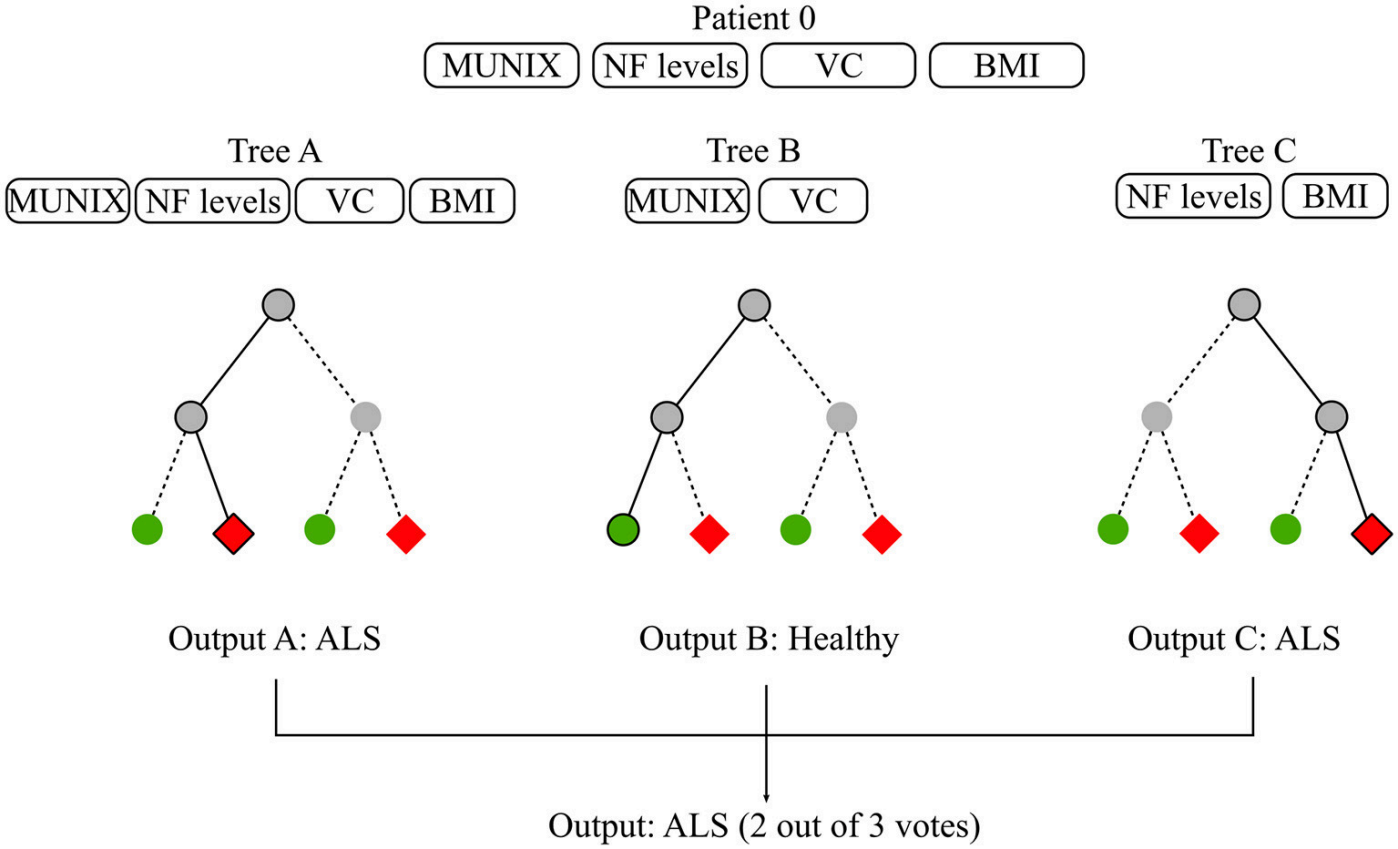
Random forest for diagnosis. The available data consist of basic biomarkers features which are MUNIX, CSF Neurofilament (NF) levels, Vital Capacity (VC), and BMI. The objective is to classify subjects between healthy and ALS patients. The RF contains 3 decisions trees which use different feature subsets to learn a diagnosis model. Tree A learns on all available features, Tree B learns on MUNIX and VC, Tree C learns on NF levels and BMI. Each tree proposes a diagnosis. RF diagnosis is computed based on the majority vote of each of the trees contained in the forest. Given that two out of three trees concluded that patient 0 had ALS, the final diagnosis suggested by the model is ALS.

“Random Forest” (RF) is a ensemble method based on decision trees. By relying on multiple learning algorithms to combine their results, ensemble methods obtain a more efficient prediction model. Each tree in the RF is built on a random subset of the training data and available features. This increases robustness to outliers and generalizability. The final estimation is the average or majority of the trees' estimation depending on whether the target is a regression or classification task (Louppe, 2014). Most RFs contain more than a hundred decision trees and decision tree length and width can also be sizable depending on the number of input features. In ML, the term “interpretability” refers to the degree to which the machine's decision is comprehensible to a human observer (Miller, 2017). While global model interpretability is de facto rather low, RFs evaluate feature importance with regards to its discriminatory power. Feature relevance is appraised based on the error function upon which the decision trees were built. Extremely Randomized Trees (Extra Trees) have shown promising results for discriminating patients suffering from Progressive Supranuclear Palsy (PSP) and Multiple System Atrophy (MSA) using speech analysis (Baudi et al., 2016). Please refer to Breiman (2001) for a more thorough description of decision trees and RFs and to Rokach (2016) and Shaik and Srinivasan (2018) for a general overview of forest models and their evolution. Figure 4 illustrates a possible diagnostic application of RF in ALS.

#### 2.2.2. Support Vector Machines

**Figure 5:**
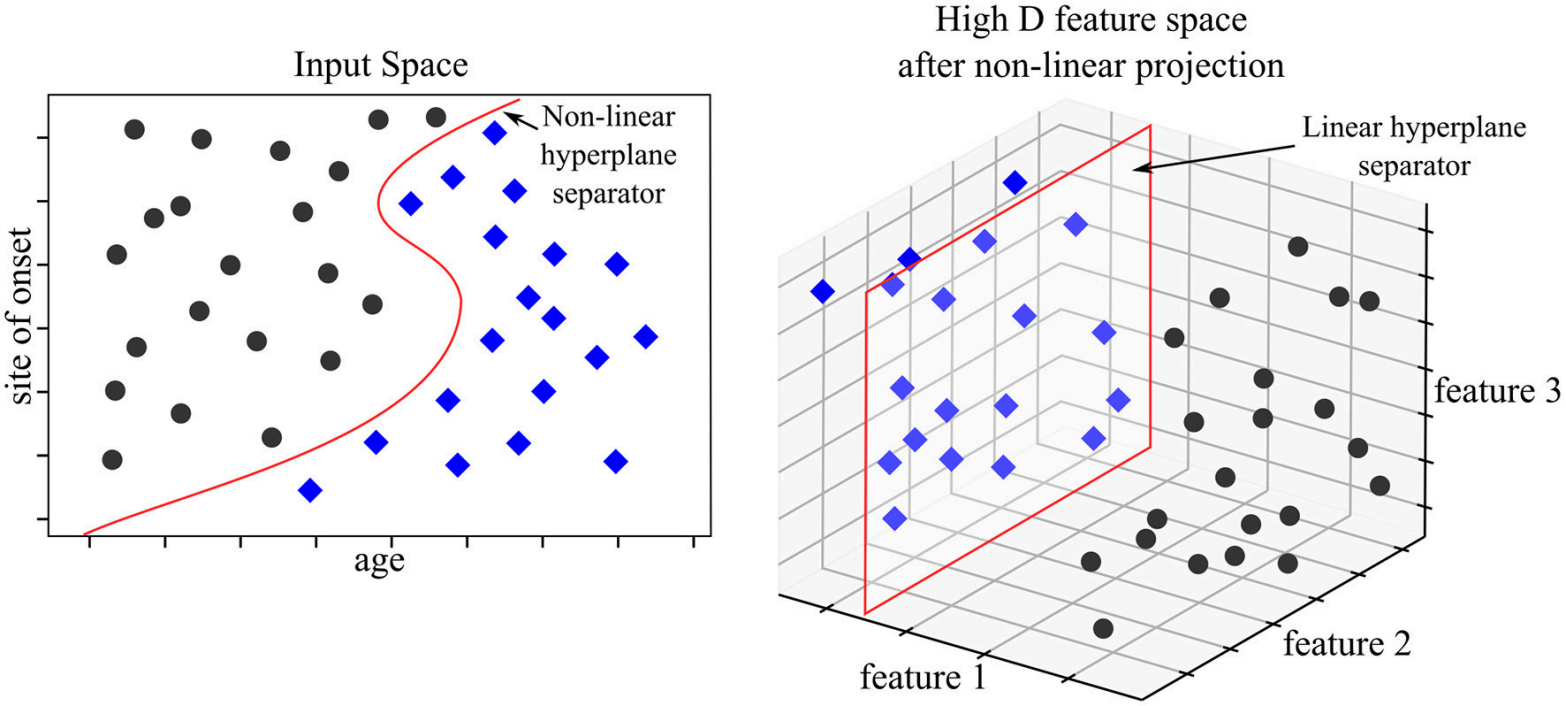
SVM model for prognosis. The available data consist of basic clinical and demographic features; age and site of onset. The objective is to classify patients according to 3-year survival. In the input space (where features are interpretable), no linear hyperplane can divide the two patient populations. The SVM model projects the data into a higher dimensional space—in our example a three dimensional space. The set of two features is mapped to a set of three features. In the feature space, a linear hyperplane can be computed which discriminates the two populations accurately. The three features used for discrimination are unavailable for analysis and interpretability is lost in the process.

Support Vector Machines (SVM) map input data into high dimensional spaces, called feature spaces, using a non-linear mapping function (Vapnik, 2000). They define a hyperplane that best separates the data. While traditional linear modeling is performed in the input space, SVMs perform linear modeling after projecting the data into another space. The features which discriminate in the projected space, also known as “feature space,” derive from input features but these are not readily interpretable. The feature space hyperplane is defined by a limited set of training points called support vectors, hence the name of the method. The chosen hyperplane maximizes the margins between the closest data samples on each side of the hyperplane, which is why SVMs are also referred to as “large margins classifier.” These vectors are identified during the “learning phase” after solving a constrained optimization problem. SVMs work as a “black box” as the logic followed by the model cannot be directly interpreted. SVM were state-of-the-art models before being outperformed by NN architecture. That being said, SVM models can adjust well to imaging specific tasks such as anomaly detection using one class SVM. Medical applications of one class SVMs have addressed the issues of tumor detection (Zhang et al., 2004) or breast cancer detection (Zhang et al., 2014). Please refer to Bishop (2016) for more information on SVMs. Figure 5 illustrates an example of a SVM used to predict prognosis in ALS.

#### 2.2.3. Neural Networks

**Figure 6:**
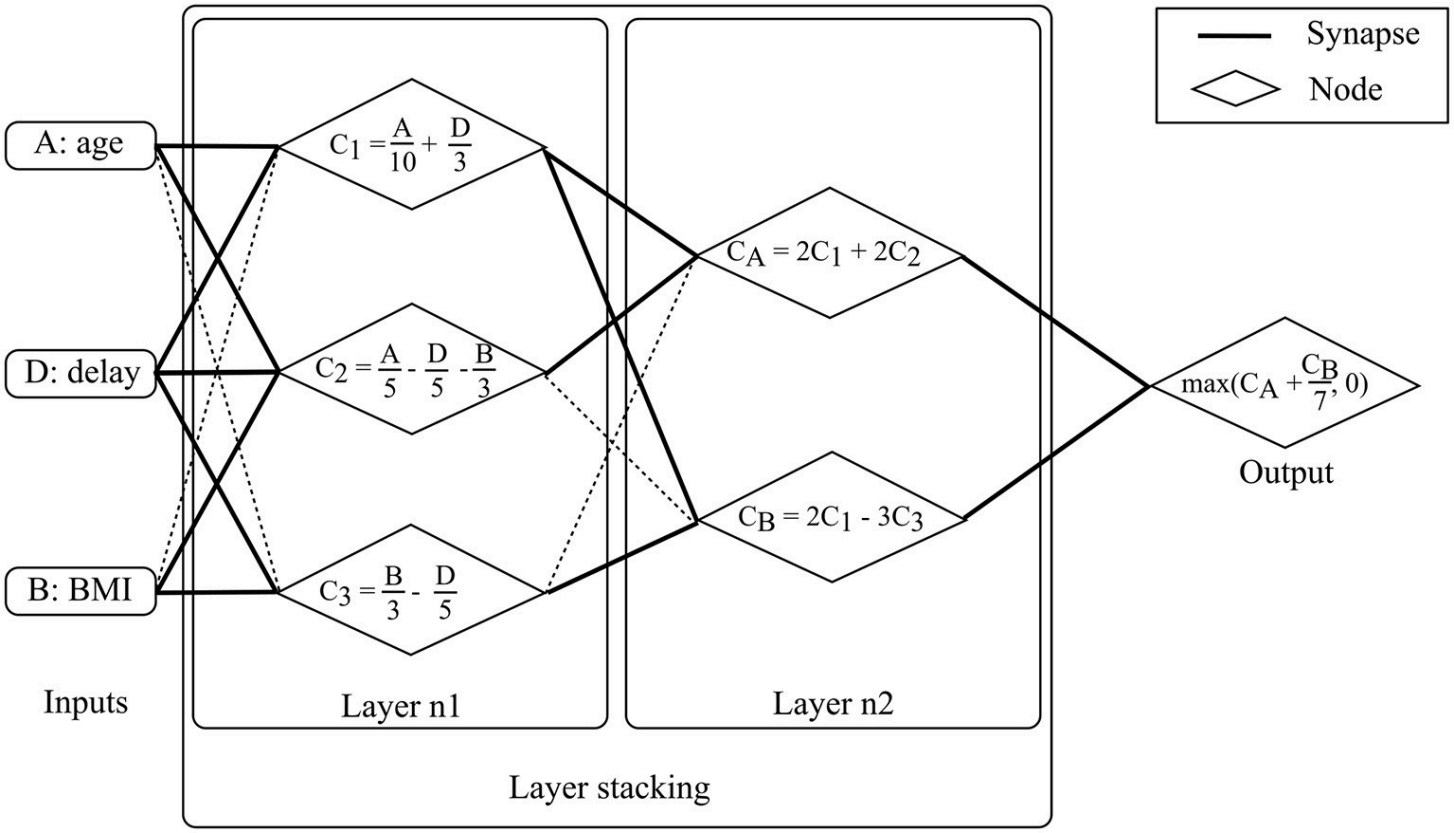
Neural Network model for prognosis. The available data consist of basic demographic and clinical features: age, BMI and diagnostic delay. For patient 0, these features are 50, 15*kg*/*m*^2^, and 15 months, respectively. The objective is to predict ALSFRS-r in 1 year. The multi-layer perceptron consists of two layers. Nodes are fed by input with un-shaded arrows. At layer 1, the three features are combined linearly to compute three node values, *C*_1_, *C*_2_, and *C*_3_. *C*_1_ is a linear combination of age and delay, *C*_2_ is a linear combination of age, delay and BMI, and *C*_3_ is a linear combination of BMI and delay. For patient 0, computing the three values returns 10, 2, and 2 for *C*1, *C*2, and *C*3, respectively. At layer 2, outputs from layer 1 (i.e., *C*_1_, *C*_2_, and *C*_3_) are combined linearly to compute two values, *C*_*A*_ and *C*_*B*_. *C*_*A*_ is a linear combination of *C*_1_ and *C*_2_ while *C*_*B*_ is a linear combination of *C*_1_ and *C*_3_. For patient 0, computing the two values gives 24 and 14 for *C*_*A*_ and *C*_*B*_, respectively. Model output is computed after computing linear combination of *C*_*A*_ and *C*_*B*_ and applying a non-linear function (in this case a maximum function which can be seen as a thresholding function which accepts only positive values). The output is the predicted motor functions decline rate. For patient 0, the returned score is 26.

A “perceptron,” also called “artificial neuron,” is a simplified representation of a human neuron. It is defined by its afferents (inputs), the inputs' respective weights and a non-linear function. The perceptron's output is the linear combination of its inputs onto which the non-linear function is applied. The linear combination consists of the sum of the multiplications of each input and their respective weight. Perceptrons can be compiled, the output of one perceptron providing the input of the next perceptron. The resulting structure is called a “multi-layer perceptron” which is the most common Neural Network (NN) framework. The contribution of each input to the neuron is modulated by its respective weight which is commonly regarded as a “synapse.” NN structures are chosen based on manual tuning and model weights are selected using iterative optimization methods. The stochastic gradient descent method is one of the most popular approaches. Specific model architectures are optimally-suited for specific data types such as “Recurrent NNs” (RNN) for time series or “Convolutional NNs” (CNN) for images. Deep learning models are NN models with significant depth or number of layers (hence the name deep learning) and extensive height or number of nodes per layer, which strongly limits their direct interpretability, similarly to SVMs. Deep learning models are currently state-of-the-art in multiple domains, specifically those which deal with imaging data. Substantial achievements were reached in the field of oncology with regards to melanoma (Esteva et al., 2017), breast cancer and prostate cancer detection (Litjens et al., 2016). Advanced neural network architecture such as the Generative Adversarial Networks (GAN) (Goodfellow et al., 2014) have been tested in a medical imaging synthesis (Nie et al., 2017) or patient record generation (Choi et al., 2017) contexts. Please refer to Goodfellow et al. (2017) for additional material on NNs, Amato et al. (2013) for NN applications in medical diagnosis, Lisboa and Taktak (2006) for NN models in decision support in cancer and Suzuki (2017). Figure 6 provides a schematic example of NNs to aid prognostic modeling in ALS using a two layer multi-layer perceptron.

#### 2.2.4. Gaussian Mixture Models

Gaussian Mixture Models (GMM) are probabilistic models which can be used in supervised or unsupervised learning. The model hypothesis is that the data can be modeled as a weighted-sum of finite Gaussian-component densities. Each density component is characterized by two parameters: a mean vector and a covariance matrix. Component parameters are estimated using the “Expectation Maximization” (EM) algorithm based on maximizing the log likelihood of the component densities. Inference is performed by drawing from the estimated mixture of Gaussian densities. GMM has achieved good results in medical applications, including medical imaging (de Luis-García et al., 2011) and diagnosing of PD (Khoury et al., 2019). Please refer to Rasmussen (2005) for additional material on GMMs, Moon (1996) for more information on the EM algorithm and Roweis and Ghahramani (1999) for a global overview of Gaussian mixture modeling.

#### 2.2.5. k-nearest Neighbors

k-Nearest Neighbors (k-NN) is an instance-based model. Inference is performed according to the values of its nearest neighbors. The advantage of the model is that limited training is required: all of the training data is kept in memory and is used during the prediction phase. Based on a selected distance function, the K most similar neighbors to the new sample are identified. The new sample's label is the average of its nearest neighbors' label. An advanced version of the method is called Fuzzy k-NN (Fk-NN) which has been used to diagnose PD based on computational voice analyses (Chen et al., 2013). Please refer to Bishop (2016) for more information on k-NN models and Aha et al. (1991) for a review on instance-based ML models.

#### 2.2.6. Boosting Methods

Boosting algorithms are ensemble methods: they rely on a combination of simple classifiers. In contrast to RF models, which are made up of decision trees and output a result based on the average or majority vote of the decision belonging to the RF mode, boosting algorithms are based on simple classifiers. The concept behind boosting is combining multiple “weak” (performance wise) learning models. This combination provides a more robust model than working with a simple base model. Model learning is based on finding the right weighting of the weak learners which make up the model to learn an efficient global model. Recent applications of boosting models include analysis of genetic information to inform on breast cancer prognosis (Lu et al., 2019) and cardiac autonomic neuropathy (Jelinek et al., 2014). Please refer to Bishop (2016) for more information on boosting methods and (Schapire, 2003) for a general overview of boosting methods.

As opposed to relying on a single ML model, models have been increasingly used in combination. For example, NN has been combined with a RF in Beaulieu-Jones and Greene (2016) where the NN output is fed into the RF model. Learning sub-models on specific feature sets have been used to feed sub-model outputs to another ML model as in Fratello et al. (2017) which trained two RF models on different imaging data sets (functional and structural MRI features) and combined intermediate outputs as the final model output. Model combination and model integration can significantly enhance overall performance, but the complexity of both approaches is often underestimated. ML model constraints are even more stringent when used as part of combined or integrated models.

### 2.3. The Limitations of Machine Learning Approaches

ML models have considerable advantages over traditional statistical approaches for modeling complex datasets. Most ML models, including the six approaches presented above, do not require stringent assumptions on data characteristics. They offer novel insights by identifying statistically relevant correlations between features and, in the case of supervised learning, of a specific outcome. Despite the pragmatic advantages, the application of ML models requires a clear understanding of what determines model performance and the potential pitfalls of specific models. The most common shortcomings will be discussed in the following section. Concerns regarding data analyses will be examined first, which include data sparsity, data bias, and causality assumptions. Good practice recommendations for model design will then be presented, including the management of missing data, model overfitting, model validation, and performance reporting.

#### 2.3.1. Data Sparsity

“Data sparsity” refers to working and interpreting limited data sets which is particularly common in medical applications. Medical data is often costly, difficult to acquire, frequently require invasive (biopsies, spinal fluid), uncomfortable (blood tests), or time consuming procedures (Magnetic Resonance Imaging). Other factors contributing to the sparsity of medical data include strict anonymization procedures, requirements for informed consent, institutional, and cross-border data management regulations, ethics approvals, and other governance issues. The processing, storage, and labeling of medical data is also costly and often requires specific funding to upkeep registries, DNA banks, brain banks, biofluid facilities, or magnetic resonance repositories (Turner et al., 2011; Bede et al., 2018b; NEALS Consortium, 2018; Neuroimaging Society in ALS, 2018). Multicenter protocols are particularly challenging and require additional logistics, harmonization of data acquisition, standardized operating procedures, and bio-sample processing, such as cooling, freezing, spinning, staining, etc.

Most ML models have originally been intended, developed, and optimized for huge quantities of data. Accordingly, the generalizability of most ML models depends heavily on the number of samples upon which it can effectively learn. Additionally, there is the “curse of dimensionality.” The number of samples required for a specific level of accuracy grows exponentially with the number of features (i.e., dimensions) (Samet, 2006). If the number of samples is restrictively low, then the features lose their discriminating power, as all samples in the dataset seem very distinct from one another (Pestov, 2007). ML models learn the underlying relationship between data samples through feature correlations. This requires the ability to discriminate between similar and dissimilar samples in the dataset. Calculating the Sample to Feature Ratio (SFR), i.e., the number of samples available per feature, is a simple way to assess whether the sample size is satisfactory for a given model. An “SFR” of around 10–15 is often considered the bare minimum (Raudys, 2001), but this is based on historical statistical models and may be insufficient for working with complex ML models. Working with a low SFR can lead to both model “underfitting” or “overfitting.” These concepts will be introduced below.

#### 2.3.2. Data Bias

Discussing data bias is particularly pertinent when dealing with medical data. Most ML models assume that the training data used is truly representative of the entire population. The entire spectrum of data distribution should be represented in the training data, just as observed in the overall population, otherwise the model will not generalize properly. For example, if a model is presented with a phenotype which was not adequately represented in the training data set, the model will at best label it as an “outlier” or at worst associate it to the wrong category label. Medical data are particularly prone to suffer from a variety of data biases which affect recorded data at different analysis levels (Pannucci and Wilkins, 2010). The four most common types of bias include: study participation bias, study attrition bias, prognostic factor measurement bias, and outcome measurement bias (Hayden et al., 2013). In ALS, study participation bias, -a.k.a. “clinical trial bias,” is by far the most significant. It affects prognostic modeling in particular, as patients in clinical trials do not reflect the general ALS population: they are usually younger, tend to suffer from the spinal form of ALS and have longer survival (Chio et al., 2011). Unfortunately, very little can be done to correct for participation bias *post-hoc*, therefore its potential impact needs to be carefully considered when interpreting the results. Study attrition bias also influences ALS studies as data censoring is not always systematically recorded. “Censoring” is a common problem in medical research; it refers to partially missing data, typically to attrition in longitudinal studies. Prognostic factor measurements can be influenced by subjective and qualitative medical assessments and by “machine bias” in imaging data interpretation. The single most important principle to manage these factors, especially if limited data are available, is overtly discussing the type of bias affecting a particular study, and openly reporting them.

#### 2.3.3. Causality Assumption

ML models identify strong (i.e., statistically significant) correlations between input features and the output in the case of supervised learning. Models can only capture observed correlations which are fully contained within the training data. Causality between features and the output cannot be solely established based on significant correlations in the dataset, especially when working with small and potentially unrepresentative population samples. Causality is sometimes inferred based on ML results which can be misleading.

### 2.4. Good Practice Recommendations

#### 2.4.1. Feature Selection

Identifying the most appropriate features is a crucial step in model design. In “sparse data” situations, the number of features should be limited to achieve an acceptable SFR and to limit model complexity. Various feature selection and engineering approaches exist, which can be chosen and combined depending on primary study objectives. It can be performed manually based on a priori knowledge or using a RF model which ranks data features based on feature importance. This method is commonly used in medical contexts as it easily gives a broad overview of the feature set. Dimension reduction is another option, with linear methods such as Principal Component Analysis (PCA) or Independent Component Analysis (ICA) and non-linear methods such as manifold learning methods. Automated feature selection methods, such as the “wrapper” or “filtering,” undergo an iterative, sometimes time-consuming process where features are selected based on their impact on overall model performance. Finally, provided that sufficient data are available, NN Auto Encoders (AE) models can also reliably extract relevant features. To this day, feature selection and engineering cannot be fully automated and human insight is typically required for manual tuning of either the features or the algorithms performing feature selection. Please refer to Guyon et al. (2006) for further information on feature selection strategies, Fodor (2002) for an overview of dimension reduction techniques and (Lee and Verleysen, 2007) for additional material on non-linear dimension reduction.

#### 2.4.2. Missing Data Management

While most ML models require complete data sets for adequate learning, medical data are seldom complete and missing features are also common. Missing data may originate from data censoring in longitudinal studies or differences in data acquisition. One common approach to missing data management is the discarding of incomplete samples. This has no effect on model design provided there is sufficient data left and that sample distribution is unaltered after discarding. This strategy usually requires large volumes of data with only a small and random subset of missing records. This condition however is rarely met in a clinical setting, where data is sparse, and missing data patterns are typically not random. Missing data can often be explained by censoring or specific testing procedures. Discarding data in these situations may increase data bias as it alters the sample distribution. The first step to missing data management is therefore to explore the mechanisms behind missing data features. Features can be “missing completely at random,” without modifying the overall data distribution, “missing at random,” when missing feature patterns are based on other features available in the dataset or “non-missing at random” for the remaining cases. Depending on the type of missing data, an appropriate imputation method should be selected. Basic data imputation methods, such as mean imputation, work well on “missing completely at random” cases but induce significant bias for “missing at random” scenarios. In this case, advanced imputation methods such as “Multiple Imputation using Chained Equations” (MICE) (van Buuren, 2007) or “Expectation Maximization” (EM) (Nelwamondo et al., 2007) algorithms operate well. Recently, missing data imputation has been managed using Denoising Auto-Encoders (DAE) models (Nelwamondo et al., 2007; Costa et al., 2018), which have a specific NN architecture. MICE and EM algorithms are statistical methods which substitute missing feature values with feature values from the most similar records in the training set. DAE models build a predictive model using the data available with no missing features to assess substitution values.“Non-missing at random” patterns are usually dealt with missing at random imputation methods, but this induces bias in data which needs to be specifically acknowledged. Please refer to Little (2002) for general principles on missing data management and (Rubin, 1987) for missing data imputation for “non-random missing” patterns.

#### 2.4.3. Model Overfitting

Each model design is invariably associated with a certain type of error. “Bias” refers to erroneous assumptions associated with a model, i.e., certain interactions between the input and the output may be overlooked by the model. ‘Variance’ refers to errors due to the model being too sensitive to training data variability. The learnt model may be excessively adjusted to the training data and poorly generalizable to the overall population if it has only captured the behavior of the training dataset. “Irreducible error” is inherent to model design and cannot be dealt with *post-hoc*. “Bias” and “variance” are interlinked, which is commonly referred to as the “bias-variance trade-off.” A high level of bias will lead to model “underfitting,” i.e., the model does not represent adequately the training data. A high level of variance will lead to model “overfitting,” i.e., the model is too specific to the training data. Overfitting is critical, as it is easily overlooked when evaluating model performance and with the addition of supplementary data, the model will not be able to accurately categorize the new data. This severely limits the use of “overfitted” models. Complex models tend to “overfit” more than simpler models and they require finer tuning. Carefully balancing variance and bias is therefore a key requirement for ML model design. Please refer to Bishop (2016) for more information on overfitting.

#### 2.4.4. Validation Schemes

Working with an optimal validation scheme is crucial in ML. Validation schemes usually split available data into “training” and “testing” datasets, so that performance can be assessed on novel data. Training and testing data should share the same distribution profile, which in turn should be representative of the entire population. Overfitting is a common shortcoming of model designs and carefully chosen validation schemes can help to avoid it. Several validation frameworks exist, “hold out validation” and “cross validation” being the two most popular. The former splits the initial dataset into two sets, one for training the other for testing. The latter performs the same splitting but multiple times. The model is learned and tested each time and the overall performance is averaged. Nevertheless, caution should be exercised in a sparse data context as validation schemes do not compensate well for poorly representative data. Please refer to Bishop (2016) for additional considerations regarding validation schemes.

#### 2.4.5. Harmonization of Performance Evaluation and Reporting

Formal and transparent performance assessments are indispensable to compare and evaluate in ML frameworks. To achieve that, standardized model performance metrics are required. In classification methods, model evaluation should include sensitivity and specificity, especially in a diagnostic context. Sensitivity (or “recall”) is the true positive rate, and specificity is the true negative rate. “Accuracy” and Area Under the “Receiver Operating Curve” (ROC) metrics can be added but should never be used alone to characterize model performance. Accuracy is the average of sensitivity and specificity. ROC is used to represent sensitivity and specificity trade-offs in a classifier model (Fawcett, 2004). The ROC space represents the relationship between the true positive rate (i.e., sensitivity) and the false positive rate (which is 1 - specificity). Given a threshold sensitivity rate, the prediction model will return a specificity rate, adding a data point to the ROC. Multiple thresholding enables the generation of the ROC curve. Perfect predictions lead to 100% sensitivity and 100% specificity (i.e., 0% false positives) which leads to an Area Under the ROC (AUC) of 1. Random predictions will return a 50% accuracy rate which is represented by a continuous straight line connecting the plot of 0% sensitivity with 100% specificity and the plot of 100% sensitivity with 0% specificity, which leads to an AUC of 0.5. Accuracy can hide a low specificity rate if there is a class imbalance and AUC can be misleading as it ignores the goodness of fit of the model and predicted probability values (Lobo et al., 2008). In regression approaches, Root Mean Squared Error (RMSE) (also referred to as Root Mean Square Deviation) and *R*^2^, the coefficient of determination, are good metrics. *R*^2^ represents the ratio of explained variation over the total variation of the data (Draper and Smith, 1998). The closer this index is to one, the more the model explains all the variability of the response data around its mean. Hence the model fits the data well. It is advisable to report multiple performance index for model evaluation as each metric reflects on a different aspect of the model. Using confidence intervals when possible is another good practice, as it conveys the uncertainty relative to the achieved error rate. General reporting guidelines for model design and model evaluation are summarized in the Transparent Reporting of a multivariate prediction model for Individual Prognosis or Diagnosis, or TRIPOD, statement (Moons et al., 2015).

Both “supervised” and “unsupervised” learning approaches have a role in clinical applications, the former for diagnosis and prognosis, the latter for patient stratification. There are a large number of ML models available, but recent work in medicine has primarily centered on three models: RF, SVM, and NN models. The advantages and drawbacks of the specific models are summarized in Table 1 (Hastie, 2003). The following factors should be considered when implementing ML models for a specific medical project:

Data limitation considerations:

– SFR assessment
– Data bias assessment
– Causality assumptions

Model design considerations:

– Feature selection with regards to SFR
– Missing data management
– Overfitting risk assessment
– Validation framework selection
– Performance metric selection
– Comprehensive model performance reporting.

**Table 1:** Overview of model pros & cons, updated from Hastie (2003).

**Characteristics**	**Neural network**	**SVM**	**Decision tree**	**RF**	**Generelized linear model**	**Gaussian mixture model**	**k-NN**	**Boosting**
Model complexity	High	High	Low	Fair	Low	High	Low	Fair
Sensitivity to dataraji sparsity	High	High	Low	Fair	Low	High	High	Fair
Sensitivity to data bias	High	High	High	High	High	High	High	High
Interpretability	Poor	Poor	Fair	Poor	Good	Poor	Good	Poor
Predictive power	Good	Good	Poor	Good	Poor	Good	Poor	Good
Ability to extract linear combinations of features	Good	Good	Poor	Poor	Poor	Poor	Poor	Poor
Natural handling ofraji missing values	Poor	Poor	Good	Good	Poor	Good	Good	Good
Robustness to outliers in input space	Poor	Poor	Good	Good	Fair	Good	Good	Good
Computational scalability	Poor	Poor	Good	Good	Good	Poor	Poor	Good

*SVM, Support Vector Machine; RF, Random Forest; k-NN, k-Nearest Neighbors*.

## 3. Results

Diagnostic, prognostic, and risk stratification papers were systematically reviewed to outline the current state of the art in ML research efforts in ALS. Consensus diagnostic criteria, established monitoring methods, and validated prognostic indicators provide the gold standard to which emerging ML applications need to be compared to.

### 3.1. Current Practices in ALS

#### 3.1.1. Current Practices in ALS for Diagnosis

The diagnosis of ALS is clinical, and the current role of neuroimaging, electrophysiology, and cerebrospinal fluid (CSF) analyses is to rule out alternative neurological conditions which may mimic the constellation of symptoms typically associated with ALS. Patients are formally diagnosed based on the revised El Escorial criteria (Brooks, 1994; Brooks et al., 2000; de Carvalho et al., 2008) which achieve low false negative rates (0.5%), but suffer from relatively high false positive rates (57%) (Goutman, 2017). As most clinical trials rely on the El Escorial criteria for patient recruitment, erroneous inclusions cannot be reassuringly ruled out (Agosta et al., 2014). Additionally, misdiagnoses are not uncommon in ALS (Traynor et al., 2000) and these, typically early-stage, ALS patients may be left out from pharmaceutical trials.

#### 3.1.2. Established Prognostic Indicators

Providing accurate prognosis and survival estimates in the early-stage ALS is challenging, as these are influenced by a myriad of demographic, genetic and clinical factors. There is a growing consensus among ALS experts that the most important determinants of poor prognosis in ALS include, bulbar-onset, cognitive impairment, poor nutritional status, respiratory compromise, older age at symptom onset, and carrying the hexanucleotide repeat on *C9orf72* (Chiò et al., 2009). Functional disability is monitored by the revised ALS Functional Rating Scale (ALSFRS-r) worldwide (Cedarbaum et al., 1999), which replaced the AALS scale (Appel ALS) (Appel et al., 1987). The ALSFRS-r is somewhat subjective as it is based on reported abilities in key domains of daily living, such as mobility, dexterity, respiratory and bulbar function. Despite its limitations, such as being disproportionately influenced by lower motor neuron dysfunction, the ALSFRS-r remains the gold standard instrument to monitor clinical trials outcomes. Prognostic modeling in ALS is typically approached in two ways; either focusing on survival or forecasting functional decline.

#### 3.1.3. Current Practices in ALS for Patient Stratification

Current patient stratification goes little beyond key clinical features and core phenotypes. These typically include sporadic vs. familial, bulbar vs. spinal, ALS-FTD vs. ALS with no cognitive impairment (ALSnci) (Turner et al., 2013). A number of detailed patient classification schemes have been proposed based on the motor phenotype alone, as in Mora and Chiò (2015) and (Goutman, 2017): “classic,” “bulbar,” “flail arm,” “flail leg,” “UMN-predominant,” “LMN-predominant,” “respiratory-onset,” “PMA,”“PLS,” “Mills' syndrome,” etc. Patients may also be classified into cognitive phenotypes such as ALS with cognitive impairment (ALSci), ALS with behavioral impairment (ALSbi), ALS-FTD, ALS with executive dysfunction (ALSexec) (Phukan et al., 2011), as presented in Figure 1. Diagnostic criteria for these phenotypes tend evolve, change and are often revisited once novel observations are made (Strong et al., 2017). Irrespective of the specific categorization criteria, these classification systems invariably rely on clinical evaluation, subjective observations, choice of screening tests, and are subsequently susceptible to classification error (Goutman, 2017). Adhering to phenotype definitions can be challenging, as performance cut-offs for some categories, such as cognitive subgroups (i.e., ALSbi/ ALSci) may be difficult to implement (Strong et al., 2009; Al-Chalabi et al., 2016). Al-Chalabi et al. (2016) used muscle bulk, tone, reflexes, age at onset, survival, diagnostic delay, ALSFRS-r decline, extra-motor involvement, symptom distribution, and family history as key features for patient stratification. ALS and FTD share common aetiological, clinical, genetic, radiological and pathological features and the existence of an ALS-FTD spectrum is now widely accepted. Up to 15% of patients develop frank dementia (Kiernan, 2018) and 60% show some form of cognitive or behavioral impairment (Phukan et al., 2011; Elamin et al., 2013; Kiernan, 2018). The presence of cognitive impairment is hugely relevant for machine-learning applications because neuropsychological deficits have been repeatedly linked to poorer survival outcomes (Elamin et al., 2011), increased caregiver burden (Burke et al., 2015), specific management challenges (Olney et al., 2005), and require different management strategies (Neary et al., 2000; Hu et al., 2009).

##### Clinical staging systems

One aspect of patient stratification is to place individual patients along the natural history of the disease by allocating them to specific disease phases or “stages.” The utility of staging in ALS is 2-fold; it guides the timing of medical interventions (non-invasive ventilation, gastrostomy, advance directives) and also allows the separation of patients early in their disease trajectory from “late-phase” patients in clinical trials. Three staging systems have been recently developed; Kings' (Roche et al., 2012), MiToS (Chiò et al., 2013a), and Fine Till 9 (FT9) (Thakore et al., 2018). While the MiToS stage can be directly calculated based on ALSFRS-r scores, the Kings' stage is a derived measure. It is noteworthy, that the stages and the ALSFRS-r score are highly correlated (Balendra et al., 2014a). Both staging systems have been cross-validated, compared and they are thought to reflect on different aspects of the disease (Hardiman et al., 2017). The MiToS system is more sensitive to the later phases of the disease, while Kings' system reflects more on the earlier phases of ALS. The FT9 system is not partial to earlier or later stages. The FT9 framework defines stages based on ALSFRS-r subscores, using 9 as a threshold after testing different values on the PRO-ACT dataset. One of the criticism of MiToS, is that stage reversion is possible and that it does not directly capture disease progression (Balendra et al., 2014b). Ferraro et al. (2016) compared MiToS and King clinical staging systems and Thakore et al. (2018) compared all three systems on PRO-ACT data.

Current diagnostic approaches in ALS are suboptimal and often lead to considerable diagnostic delay. Prognostic protocols are not widely validated and current patient stratification frameworks don't represent the inherent heterogeneity of ALS. Accordingly, machine-learning approaches have been explored to specifically address these three issues.

### 3.2. Results in Diagnosis

#### 3.2.1. Advances in Biomarker Research

The majority of ML research projects focus on the development, optimization, and validation of diagnostic biomarkers. These typically include clinical, biofluid, and neuroimaging indicators. Diagnostic model performance depends on the feature's ability to describe how the disease affects the subjects. Optimal diagnostic biomarkers should not only discriminate between ALS patients and healthy controls but also between ALS patients and patients with mimic or alternative neurological conditions (Bede, 2017). Ideally, an optimal diagnostic model should have outstanding early-stage sensitivity and specificity so that patients can be recruited into clinical trials early in their disease.

##### Clinical biomarker research

MUNIX (Fathi et al., 2016) is a non-invasive neurophysiological method which is extensively used in both clinical and research settings. It may also have the ability to capture pre-symptomatic motor neuron loss (Escorcio-Bezerra et al., 2018), therefore it has the potential to confirm early-stage disease in suspected cases. An earlier diagnosis would in turn enable the earlier initiation of neuroprotective therapy with established drugs and more importantly, earlier entry into clinical trials.

##### Biological biomarker research

Cerebrospinal Fluid (CSF) Neurofilaments (NF) are regarded as one of the most promising group of “wet” biomarkers in ALS (Rossi et al., 2018; Turner, 2018). Typically, research studies assess both Neurofilament Light (NF-L) chain and phosphorylated Heavy (pNF-H) chain levels that are released due to axonal degeneration and can be detected in the CSF and serum. Studies have consistently shown increased CSF pNF-H levels in ALS and up to ten times higher levels than in patients with Alzheimer disease (Brettschneider et al., 2006) or other neurological conditions (Gresle et al., 2014; Steinacker et al., 2015). Even though ALS studies have consistently detected raised pNF-H concentrations, these values vary considerably in the different reports. CSF NF-L levels were linked to reduced pyramidal tract Fractional Anisotropy (FA) and increased Radial Diffusivity (RD) (Menke et al., 2015) and NF-L levels are also thought to correlate with progression rates (Tortelli et al., 2014). Other biological biomarkers include proxies of oxidative stress, such as CSF 4-hydroxy-2,3-nonenal (4-HNE) (Simpson et al., 2004) or 3-nitrotyrosine (3-NT) (Tohgi et al., 1999). Neuroinflammation is another important feature of ALS, and several studies have detected an increase in inflammation-associated molecules, such as interleukin-6 (IL-6) and TNF alpha (*TNF* − α) (Moreau et al., 2005) and galectin-3 (GAL-3) (Zhou et al., 2010). Increased levels of CSF Chitotriosidase-1 (CHIT1) is thought to indicate increased microglial activity (Varghese et al., 2013). Raised levels of CSF hydrogen sulfide (*H*_2_*S*) was also reported in ALS, which is released by astrocytes and migrolia and is known to be toxic for motor neurons (Davoli et al., 2015). These are all promising wet biomarkers, indicative of disease-specific pathological processes and it is likely that a panel of several biomarkers may be best suited for diagnostic purposes.

##### Genetic biomarker research

A shared pathological hallmark of neurodegenerative conditions is protein aggregation. The accumulation of the Transactive Response DNA Binding Protein 43 (*TDP-43*) is the most consistent pathological finding in approximately 95% of ALS cases (Neumann et al., 2006). Given the widespread aggregation and accumulation of *TDP-43* in FTD-ALS spectrum, *TDP-43* detection, measurement or imaging is one of the most promising biomarkers strategies. A recent meta-analysis evaluated the diagnostic utility of CSF *TDP-43* levels in ALS (Majumder et al., 2018) and found that increased levels may be specific to ALS, as *TDP-43* levels are significantly raised compared to FTD as well. Reports on *SOD1* levels in the CSF of ALS patients have been inconsistent; some studies detected increased levels (Kokić et al., 2005) whereas others have identified decreased levels (Ihara et al., 2005) or levels comparable to controls (Zetterström et al., 2011).

##### Proteomics biomarker research

Beyond the interpretation of clinical and imaging data, ML models have an increasing role in genetics, RNA processing and proteomics (Bakkar et al., 2017). Using IBM Watson 5 new RNA-Binding Proteins (RBPs) were identified which were previously not linked to ALS; Heterogeneous nuclear ribonucleoprotein U (*hnRNPU*), Heterogeneous nuclear ribonucleoprotein Q (*SYNCRIP*), Putative RNA-binding protein 3 (*RBMS3*), ell Cycle Associated Protein 1 (*Caprin-1*) and Nucleoporin-like 2 (*NUPL2*). ML models play an important role in modern genetic analyses (Libbrecht and Noble, 2015) but considerable variations exist in their application between various medical subspecialties. One of the roles of ML in genomics is to identify the location of specific protein-encoding genes within a given DNA sequence (Mathé et al., 2002). In the field of proteomics, ML has been extensively utilized to predict 3-dimensional folding patterns of proteins. Approaches such as Deep Convolutional Neural Fields (DeepCNF) have been successful in predicting secondary structure configurations (Wang et al., 2016). In proteomics, ML models are also utilized for loop modeling, and protein side-chain prediction (Larranaga et al., 2006).

##### Imaging biomarker research

Neuroimaging offers unique, non-invasive opportunities to characterize disease-associated structural and functional changes and imaging derived metrics have been repeatedly proposed as candidate biomarkers (Turner et al., 2011; Agosta et al., 2018a; Bede et al., 2018b). The primary role of MRI in current clinical practice is the exclusion of alternative structural, neoplastic and inflammatory pathology in the brain or spinal cord which could manifest in UMN or LMN dysfunction similar to ALS. Diffusion tensor imaging (DTI) has gained a lot of attention as DTI-derived metrics, such as FA, Mean Diffusivity (MD), RD, or Axial Diffusivity (AD) have already been successfully used to identify ALS patients in ML models (RF) (Bede et al., 2017; Querin et al., 2018). The DTI signature of ALS is firmly established thanks to a myriad of imaging studies, and it includes the commissural fibers of the corpus callosum and the bilateral Corticospinal Tract (CST) (Turner et al., 2009; Bede et al., 2014). The latter has been associated to clinical UMN dysfunction, as well as rate of progression in specific sub-regions (Schuster et al., 2016a). White matter degeneration in frontal and temporal regions have been linked to cognitive and behavioral measures (Agosta et al., 2010; Christidi et al., 2017) and specific genotypes (Bede et al., 2013a). While callosal (Filippini et al., 2010; Bede et al., 2013a) and CST (Agosta et al., 2018b) degeneration seems to be a common ALS-associated signature, frontotemporal and cerebellar white matter degeneration seems to be more specific to certain phenotypes (Prell and Grosskreutz, 2013; Bede et al., 2014). From a gray matter perspective, motor cortex atrophy is a hallmark finding irrespective of specific genotypes and phenotypes (Bede et al., 2012) which is readily captured by cortical thickness or volumetric measures. Other gray matter regions, such as frontal (Lulé et al., 2007), basal ganglia (Bede et al., 2013c, 2018a; Machts et al., 2015), or cerebellar regions (Prell and Grosskreutz, 2013; Batyrbekova et al., 2018) may be more specific to certain patient cohorts. What is important to note, is that considerable white matter degeneration can already be detected around the time of diagnosis which progress relatively little, as opposed to the incremental gray matter findings in the post-symptomatic phase of the disease (Bede and Hardiman, 2017; Menke et al., 2018). The relevance of these observations is that white matter metrics may be particularly suitable for diagnostic models, whereas gray matter metrics in monitoring applications.

#### 3.2.2. Overview of Research in Diagnosis

ML methods have already been extensively tested to aid the diagnosis of ALS (Gordon et al., 2009; Welsh et al., 2013; Sarica et al., 2016; Schuster et al., 2016b; Bede et al., 2017; Ferraro et al., 2017; Fratello et al., 2017; D'hulst et al., 2018; Li et al., 2018; Querin et al., 2018). Diagnostic models are typically developed within a classification framework with limited category labels, such as “healthy” vs. “ALS.” Srivastava et al. (2012) implemented a model to discriminate patients within the Spinal Muscular Atrophy (SMA) spectrum. A similar attempt has not been made in ALS yet but could prove very valuable. A number of imaging features have been explored in recent years (Sarica et al., 2016; Schuster et al., 2016b; Bede et al., 2017; Ferraro et al., 2017; Fratello et al., 2017; D'hulst et al., 2018; Querin et al., 2018).

Performance was highest using combined imaging metrics (Bede et al., 2017) outperforming diagnostic models relying solely on clinical features (Li et al., 2018) which typically achieve up to 68% sensitivity and 87% specificity. Current models however are severely limited by small sample sizes and achieve lower true positive rates than the El Escorial's criteria but dramatically improve false negative rates. In general, diagnostic models based on imaging data achieve a sensitivity above 80% which is very encouraging especially given the emergence of larger data sets (Müller et al., 2016). It is crucial to evaluate model performance in comparison to the current gold standard criteria and report both sensitivity (true positive rate) and specificity (true negative rate). Additional metrics seem also necessary such as accuracy and AUC which provides a global indication of the model's performance.

##### Performance analysis

Welsh et al. (2013), Schuster et al. (2016b), Bede et al. (2017), Ferraro et al. (2017), Fratello et al. (2017), D'hulst et al. (2018), and Querin et al. (2018) only used single-centre imaging data for their model design. Bede et al. (2017) used a canonical discriminant function and achieved an accuracy of 90% (for 90% sensitivity and 90% specificity). Sarica et al. (2016), Ferraro et al. (2017), Fratello et al. (2017), and Querin et al. (2018) used RFs achieving accuracy rates between 77.5 and 86.5%. Schuster et al. (2016b) used a binary logistic regression model and reached 78.4% (90.5% sensitivity and 62.5% specificity). Welsh et al. (2013) and D'hulst et al. (2018) used SVMs reaching an accuracy of 71 and 80%, respectively. A relatively low accuracy of 71% (Welsh et al., 2013) and low specificity of 12.5% (D'hulst et al., 2018) may stem from model overfitting. The complexity of SVM models, class imbalance (D'hulst et al., 2018), data sparsity (Welsh et al., 2013) are some of the factors which may contribute to their relatively poorer performance. Li et al. (2018) used a linear regression model based on clinical data and reached 77.5% accuracy, 68% sensitivity and 87% specificity. Half of the studies (Welsh et al., 2013; Sarica et al., 2016; Bede et al., 2017; D'hulst et al., 2018; Querin et al., 2018) focused on discriminating ALS patients from healthy controls. Four studies (Gordon et al., 2009; Ferraro et al., 2017; Fratello et al., 2017; Li et al., 2018) went further and attempted to identify ALS within a range of neurological diseases including patients with Parkinson's Disease (PD), Kennedy's Disease (KD), PLS, etc. Srivastava et al. (2012) focused on identifying specific SMA phenotypes. Please refer to Table 2 for an overview of ML papers focusing on the diagnosis of ALS.

**Table 2:** Research overview: diagnosis.

**Key**	**Dataset(s) origin**	**Dataset(s) type**	**Dataset(s) length**	**Scope**	**Biomarker(s) type**	**Pre-processing (if any)**	**Validation (if any)**	**Model(s) tested**	**Performance**
Gordon et al., 2009	Eleanor and Lou Gehrig MDA/ALS Research Center	Real-life	34	ALS, UMN, PLS	Clinical	FS	None described	Linear regression	-
Srivastava et al., 2012	Boston Children Hospital	Real-life	46	SMA phenotypes	Clinical, genetic	FS	CV	SVM	AUC (0.928)
Welsh et al., 2013	Michigan MND Clinic	Real-life	63	ALS, healthy	Imaging	FS	LOOV	SVM	AUC:0.7, Acc:71%, Spec:74%, Sens:68.8%
Sarica et al., 2016	Catanzaro Magna Graecia University	Real-life	48	ALS, healthy	Clinical, imaging	SP, FS	CV	RF	Acc:80%
Schuster et al., 2016b	Trinity College Dublin	Real-life	147	ALS, healthy	Imaging	SP, FS	CV	Logistic regression	Acc:78.4%, raji Sens:90.5%, rajiSpec:62.5%
Bede et al., 2017	Trinity College Dublin	Real-life	150	ALS, healthy	Imaging	SP, FS	HOV	Discriminant function	Acc:90%, Sens:90%, Spec:90%
Ferraro et al., 2017	MND Clinics in Northern Italy	Real-life	265	ALS, UMN, ALS mimics	Imaging	SP	HOV	RF	Acc:87%, Spec:75%, Sens:92%
Fratello et al., 2017	UK PD Brain Bank	Real-life	120	ALS, PD, healthy	Imaging	SP, FS	CV	RF	Acc:80%
D'hulst et al., 2018	University Hospital rajiLeuven and Turino ALS Center	Real-life	370	ALS, healthy	Imaging	SP	LOOV	SVM	Acc:80%, Sens:85%, Spec:12.5%
Li et al., 2018	Australia	Clinical trial	81	ALS, KD, ALS mimics	Clinical	FS	None described	Linear regression	Acc:77.5%, Sens:68%, Spec:87%
Querin et al., 2018	Pitiè Salpêtrière Hospital	Real-life	105	ALS, healthy	Imaging	SP	CV	RF	AUC:0.96, Acc:86.5%, Sens:88%, Spec:85%

*CV, Cross Validation; LOOV, Leave One Out Validation; HOV, Hold Out Validation; AUC, Area under the ROC Curve; Acc, Accuracy; Sens, Sensitivity; Spec, Specificity; PD, Parkinson's Disease; FS, Feature Selection; SP, Signal Processing*.

##### Technical analysis

From a methods point of view, all of the above papers overtly present their pre-processing pipeline (Sarica et al., 2016; Schuster et al., 2016b; Bede et al., 2017; Ferraro et al., 2017; Fratello et al., 2017; D'hulst et al., 2018; Querin et al., 2018) and feature selection strategy (Gordon et al., 2009; Srivastava et al., 2012; Welsh et al., 2013; Sarica et al., 2016; Schuster et al., 2016b; Bede et al., 2017; Fratello et al., 2017; Querin et al., 2018). Imaging analyses need to take the effect of age, gender, and education on MRI data into account, as these have a major impact on white and gray matter metrics. Studies control for these demographic factors differently; while age is generally adjusted for (Zhang et al., 2018), the effect of gender (Bede et al., 2013b) and education (Cox et al., 2016) are often overlooked which can affect model development. Judicious feature selection is paramount as model complexity is directly related to the number of features fed into the model. Limiting model complexity, especially in the context of sparse data is crucial to avoid model overfitting. Feature selection is often based, either on group comparisons or a priori imaging or pathological information. Features often include imaging measures of key, disease-associated anatomical regions, such as measures of the motor cortex or pyramidal tracts (Bede et al., 2016). Existing studies use very different validation schemes to test model performance. Cross-validation is the most commonly used (Srivastava et al., 2012; Sarica et al., 2016; Schuster et al., 2016b; Fratello et al., 2017; Querin et al., 2018), followed by holdout validation (Bede et al., 2017; Ferraro et al., 2017) and leave-one-out validation (Welsh et al., 2013; D'hulst et al., 2018). While robust validation schemes are essential, they don't circumvent overfitting especially when limited data are available. “Cross validation” and “leave-one-out” approaches are generally more robust than holdout validation. Special caution should be exercised with regards to validation reports in sparse data situations, where validation schemes have a limited ability to assess model performance. Querin et al. (2018) and Li et al. (2018) both show SFR higher than ten (15 and 12, respectively) which comply with minimum SFR recommendations (Raudys, 2001).

### 3.3. Results in Prognosis

#### 3.3.1. Advances in Biomarker Research

As the precise mechanisms of disease propagation in ALS are largely unknown (Ravits, 2014; Ayers et al., 2015), research has focused on the identification of candidate prognostic biomarkers including potential clinical, biological, imaging, and genetic indicators. Prognostic model performance depends on the feature's ability to capture the disease spread. Optimal prognostic biomarkers should not only discriminate between different ALS phenotypes but categorize individual patients to common disease progression rates (slow vs. fast progressors) (Schuster et al., 2015).

##### Clinical biomarker research

Several recent studies examined the specific impact of psychosocial factors, cognitive impairment, nutritional status and respiratory compromise, on prognosis. Psychosocial adjustments in ALS may have an under-recognized impact on prognosis (Matuz et al., 2015). The potential effect of mood on disease progression has only been investigated on a relatively small number of samples to date (Johnston et al., 1999).

##### Biological biomarker research

Recent research suggests that prognostic modeling that does not rely on a priori hypotheses could lead to more accurate prognostic models than does driven by pre-existing hypotheses. For instance, elevations in Creatine Kinase (CK) were linked to LMN involvement and faster disease progression (Rafiq et al., 2016; Goutman, 2017) using the PRO-ACT data (Ong et al., 2017).

##### Genetic biomarker research

In a clinical setting, genetic testing is often only performed in familial forms of ALS. *C9orf72* repeat expansions account for 40% of hereditary ALS cases and 10% of sporadic ALS cases (Goutman, 2017) and hexanucleotide repeats are associated with specific clinical traits (Byrne et al., 2012). More than 30 genes have been implicated in the pathogenesis of ALS to date and samples are often screened for Angiogenin (*ANG*), Dynactin subunit 1 (*DCTN1*), Fused in sarcoma (*FUS*), Optineurin (*OPTN*), *SOD1*, Transactive Response DNA Binding Protein (*TARDBP*), Ubiquilin (*UBQLN2*), Valosin-Containing Protein (*VCP*) (Chen et al., 2013; Renton et al., 2013; Taylor J. P. et al., 2016), Alsin Rho Guanine Nucleotide Exchange Factor (*ALS2*), Polyphosphoinositide phosphatase (*FIG4*), Probable Helicase Senataxin (*SETX*), Spatacsin (*SPG11*), Vesicle-Associated membrane protein-associated Protein B/C (*VAPB*) (Chen et al., 2013; Renton et al., 2013), Heterogeneous nuclear ribonucleoprotein A1 (*HNRNPA1*), Profilin 1 (*PFN1*), Sequestosome 1 (*SQSTM1*) (Renton et al., 2013; Taylor J. P. et al., 2016), Coiled-coil-helix-coiled-coil-helix domain-containing protein 10 (*CHCHD10*), Matrin 3 (*MATR3*), Serine/Threonine-protein Kinase (*TBK1*) (Taylor J. P. et al., 2016), sigma-1 receptor (*SIGMAR1*), Diamine oxidase (*DAO*) (Chen et al., 2013), Charged multivesicular body protein 2b (*CHMP2B*), Ataxin-2 (*ATXN2*), Neurofilament Heavy (NEFH), Elongator complex protein 3 (*ELP3*) (Renton et al., 2013) as well as Receptor tyrosine-protein kinase (*ERBB4*), Unc-13 homolog A (*UNC13A*), Peripherin (*PRPH*), TATA-binding protein-associated factor 2N (*TAF15*), Spastin (*SPAST*), Lamin-B1 (*LMNB1*), Sterile alpha and TIR motif-containing protein 1 (*SARM1*), *C21orf2*, (never in mitosis gene a)-related kinase 1 (*NEK1*), Granulin Precursor (*GRN*), Microtubule Associated Protein Tau (*MAPT*) and Presenilin 2 (*PSEN2*). IBM Watson software has been successfully utilized to identify other candidate genes; such as *hnRNPU, SYNCRIP, RBMS3, Caprin-1* and *NUPL2* (Bakkar et al., 2017). Genomic research teams have increasingly capitalized on ML methods worldwide, as they can handle copious amounts of data for systematic processing, genomic sequence annotation, DNA pattern recognition, gene expression prediction, and the identification of genomic element combinations (Libbrecht and Noble, 2015).

##### The benefit of multiparametric datasets

Early machine learning efforts have been hampered by the lack of large data sets in ALS, which is increasingly addressed by the availability of large international repositories, such as those maintained by NISALS (Müller et al., 2016; Neuroimaging Society in ALS, 2018), NEALS (NEALS Consortium, 2018), and PRO-ACT which includes more than 10 000 patient records from 23 clinical trials in total. Similar initiatives had been carried out in other neurological conditions, as part of the Alzheimer's Disease Neuroimaging Initiative (ADNI) (Mueller et al., 2005), the Parkinson's Progression Marker's Initiative (PPMI) (Marek et al., 2011) and Tract HD (Tabrizi et al., 2012). Emerging large data sets, like PRO-ACCT, also serve as validation platforms for previously identified biomarkers. For example, vital capacity was identified as early as 1993 (Schiffman and Belsh, 1993) as a predictor of disease progression and proved relevant in the Prize4Life challenge (Küffner et al., 2014). Other validated biomarkers include creatinine (Atassi et al., 2014; Küffner et al., 2014; Ong et al., 2017), BMI (Atassi et al., 2014; Küffner et al., 2014; Ong et al., 2017), CK (Ong et al., 2017), Alkaline Phosphatase (ALP)(Küffner et al., 2014; Ong et al., 2017), albumin (Ong et al., 2017), total birilubin (Ong et al., 2017), and uric acid (Atassi et al., 2014). Other predictive clinical features such as onset at age, region of onset, and respiratory compromise have long been firmly established (Chio et al., 2009; Creemers et al., 2014).

#### 3.3.2. Overview of Research in Prognosis

While prognostic forecasting has historically been undertaken using traditional statistical approaches in ALS (Ince et al., 2003; Forbes, 2004; Visser et al., 2007; Coon et al., 2011; Atassi et al., 2014; Elamin et al., 2015; Marin et al., 2015; Rong et al., 2015; Tortelli et al., 2015; Wolf et al., 2015; Knibb et al., 2016; Reniers et al., 2017), ML models have an unprecedented potential to identify novel prognostic indicators (Gomeni and Fava, 2013; Hothorn and Jung, 2014; Ko et al., 2014; Beaulieu-Jones and Greene, 2016; Taylor A. A. et al., 2016; Huang et al., 2017; Jahandideh et al., 2017; Ong et al., 2017; Schuster et al., 2017; Seibold et al., 2017; van der Burgh et al., 2017; Bandini et al., 2018; Pfohl et al., 2018; Westeneng et al., 2018). Most prognostic models use clinical features to determine prognosis in ALS but two recent papers enriched their clinical data with imaging measures (Schuster et al., 2017; van der Burgh et al., 2017). Seven studies designed their prediction model around both clinical and biological data, (Hothorn and Jung, 2014; Ko et al., 2014; Beaulieu-Jones and Greene, 2016; Huang et al., 2017; Jahandideh et al., 2017; Ong et al., 2017; Seibold et al., 2017) and nine studies developed their prognostic model based on PRO-ACT data, (Gomeni and Fava, 2013; Hothorn and Jung, 2014; Ko et al., 2014; Beaulieu-Jones and Greene, 2016; Taylor A. A. et al., 2016; Huang et al., 2017; Jahandideh et al., 2017; Ong et al., 2017; Seibold et al., 2017). Prognosis is typically defined either as functional decline or survival and is either approached as a classification problem with predefined categories or as a regression problem with a specific survival or functional thresholds. The most accurate regression approach had a RMSE of 0.52 (with regards to the ALSFRS rate) (Hothorn and Jung, 2014) and one of the most accurate classification method (Ko et al., 2014) reached 66% accuracy, 66% sensitivity, and 65% specificity using a RF. Bandini et al. (2018) achieved 87% accuracy with a SVM model a fairly complex model built on only 64 samples - which puts the model at a high risk of overfitting. For outcome prediction as a regression problem, best results were reached by Pfohl et al. (2018) using a RF. For outcome prediction as a classification problem, best performance was achieved by Westeneng et al. (2018) with 78% accuracy using a multivariate Royston-Parmar model.

##### Statistical methods

Previous prognostic studies in ALS primarily used traditional statistical approaches, mostly Cox regressions, mixed effect models and Kaplan-Meier estimators. These models have relatively stringent data assumptions which limit model validity and limit data exploration. Nevertheless, they were instrumental in identifying key prognosis indicators in ALS, such as diagnostic delay (Forbes, 2004; Elamin et al., 2015; Marin et al., 2015; Wolf et al., 2015; Knibb et al., 2016; Reniers et al., 2017), age at symptom onset (Forbes, 2004; Marin et al., 2015; Wolf et al., 2015; Knibb et al., 2016; Reniers et al., 2017), functional disability (Visser et al., 2007; Elamin et al., 2015; Marin et al., 2015; Wolf et al., 2015; Reniers et al., 2017), El Escorial categorization (Forbes, 2004; Marin et al., 2015; Wolf et al., 2015), comorbid FTD or executive dysfunction (Elamin et al., 2015; Wolf et al., 2015; Knibb et al., 2016), site of onset (Forbes, 2004; Elamin et al., 2015), Riluzole therapy (Forbes, 2004; Knibb et al., 2016), vital capacity (Visser et al., 2007), muscle weakness (Visser et al., 2007), involvement of body regions (Visser et al., 2007), gender (Wolf et al., 2015), BMI (Atassi et al., 2014), presence of *C9orf72* mutations (Reniers et al., 2017). Other prognostic studies focused on the macrophage marker Cluster of Differentiation 68 (*CD68*) (Ince et al., 2003), neuropsychological deficits (Coon et al., 2011), creatinine and uric acid levels (Atassi et al., 2014), tongue kinematics (Rong et al., 2015), anatomical spread (Tortelli et al., 2015), and LMN involvement (Reniers et al., 2017). A number of studies have specifically focused on survival (Forbes, 2004; Visser et al., 2007; Coon et al., 2011; Atassi et al., 2014; Elamin et al., 2015; Marin et al., 2015; Tortelli et al., 2015; Wolf et al., 2015; Reniers et al., 2017). Ince et al. (2003) performed an a posteriori analysis of disease progression based on MRI data. Coon et al. (2011) analyzed the impact of language deficits and behavioral impairment on survival. Rong et al. (2015) assessed the implications of early bulbar involvement. To this date, most reliable predictive features are clinical factors, but similar approaches can be extended to biofluid, genetic, and imaging data. Both ML and traditional statistical approaches perform better with multi-modal data. Existing ML studies in ALS show considerable differences in their methodology and validation approaches. Please refer to Table 3 for an overview of ALS papers focusing on prognostic modeling.

**Table 3:** Research overview: prognosis with statistical models.

**Key**	**Dataset(s) origin**	**Dataset(s) type**	**Dataset(s) length**	**Scope**	**Biomarker(s) type**	**Pre-processing (if any)**	**Validation (if any)**	**Model(s) tested**
Ince et al., 2003	Newcastle upon rajiTyne MND clinic	Real-life	81	Progression	Imaging	None described	Not required	Univariate analysis
Forbes, 2004	Scottish ALS-MND Register	Population	1226	Outcome	Clinical	None described	Not required	Cox time rajidependent regression modeling
Visser et al., 2007	Dutch university hospitals	Real-life	37	Outcome	Clinical, genetic, biological	None described	Not required	Univariate analysis
Coon et al., 2011	Mayo Clinic	Real-life	56	Outcome	Clinical, imaging	None described	Not required	KM analysis
Atassi et al., 2014	PRO-ACT	Clinical trial	8635	Outcome, progression	Clinical, biological	Data cleaning	Not required	Multivariate analysis
Elamin et al., 2015	Irish and Italian (Piemonte) ALS registry	Population	326	Outcome	Clinical, genetic	FS	HOV	Proportional hazards Cox
Marin et al., 2015	FRALim register	Population	322	Outcome	Clinical	None described	Not required	Cox regression (KM)
Rong et al., 2015	-	Clinical trial	66	Progression	Clinical	FS	Not required	Linear Mixed Effect, KM analysis
Tortelli et al., 2015	University of Bari MND Center	Clinical trial	145	Outcome	Clinical	None described	Not required	Bivariate model for correlation
Wolf et al., 2015	Rhineland-Palatinate Register	Population	193	Outcome	Clinical	FS	Not required	Cox proportional hazards
Knibb et al., 2016	South-East England Register	Population	575	Outcome, progression	Clinical	MVR	CV	Cox proportional hazards, ACT
Reniers et al., 2017	University Hospitals Leuven	Real-life	396	Outcome	Clinical	None described	Not required	Univariate and multivariate Cox regression

*HOV, Hold Out Validation; CV, Cross Validation; ACT, Accelerated Failure Time; KM, Kaplan Meier; MVR, Missing Value Removal; FS, Feature Selection*.

##### Performance analyses

RF is the most commonly used model in ALS, implemented in eight of the fourteen reviewed studies (Hothorn and Jung, 2014; Ko et al., 2014; Beaulieu-Jones and Greene, 2016; Taylor A. A. et al., 2016; Huang et al., 2017; Jahandideh et al., 2017; Seibold et al., 2017; Pfohl et al., 2018) and it is also one of the best performing methods (Beaulieu-Jones and Greene, 2016; Taylor A. A. et al., 2016; Huang et al., 2017; Pfohl et al., 2018). Boosting, another ensemble method, was tested by Jahandideh et al. (2017) and Ong et al. (2017). The boosting algorithm outperformed the RF model in Jahandideh et al. (2017). NN models were used successfully in two studies: Beaulieu-Jones and Greene (2016) and van der Burgh et al. (2017). Regression models have also been extensively used in ALS, including generalized linear models (Taylor A. A. et al., 2016; Huang et al., 2017; Pfohl et al., 2018), Royston-Parmar models for Westeneng et al. (2018), and non-linear Weibull models (Gomeni and Fava, 2013). Regression models, despite their stringent assumptions, have great potential in clinical applications (Westeneng et al., 2018). Seibold et al. (2017) used an innovative RF approach to establish the impact of Riluzole therapy on functional decline and survival. Out of the ten models built on clinical data, nine were based on PRO-ACT data (Gomeni and Fava, 2013; Hothorn and Jung, 2014; Ko et al., 2014; Beaulieu-Jones and Greene, 2016; Taylor A. A. et al., 2016; Huang et al., 2017; Jahandideh et al., 2017; Ong et al., 2017; Seibold et al., 2017).

Prognosis in ALS is typically either addressed as a classification or a regression problem. In studies using the classification approach, categories are defined based on functional decline (Gomeni and Fava, 2013; Ko et al., 2014; Ong et al., 2017; Westeneng et al., 2018), survival (Schuster et al., 2017; Pfohl et al., 2018), or disease phase (Bandini et al., 2018). Studies using the regression approach predicted survival (Beaulieu-Jones and Greene, 2016; Huang et al., 2017; van der Burgh et al., 2017; Pfohl et al., 2018), Riluzole effect (Seibold et al., 2017), functional decline (Hothorn and Jung, 2014; Taylor A. A. et al., 2016), or respiratory function (Jahandideh et al., 2017). ALSFRS-r is invariably used in these studies, highlighting that it remains the gold standard instrument to monitor disease progression. Most prognostic models rely solely on clinical features, sometimes enriched with biological data. Radiological data are seldom used in these models, and often rely on relatively small datasets; Schuster et al. (2017) included 69 and van der Burgh et al. (2017) 135 subjects. Despite their considerable sample size limitations, these models achieved relatively promising results with accuracy rates above 79%. Unfortunately, as in the case of diagnostic modeling, large datasets of imaging data, especially longitudinal, are still relatively difficult to acquire in single-centre settings.

A variety of metrics have been utilized for model performance evaluation. For classification tasks, these typically include AUC, specificity and sensitivity, accuracy and concordance (C-index), and for regression methods, RMSE, *R*^2^, mean absolute error, and Pearson correlations between real and predicted estimates are usually reported. Approximately half of the reviewed papers used RF to assess variable importance (Hothorn and Jung, 2014; Huang et al., 2017; Jahandideh et al., 2017; Ong et al., 2017; Seibold et al., 2017; Pfohl et al., 2018; Westeneng et al., 2018). Pfohl et al. (2018) carried out correlation analysis and PCA component projection analysis which proved very instructive. Gamma glutamyl-transferase, was identified as a potential prognostic indicator by Ong et al. (2017). Despite the obvious advantages, model testing is only rarely carried out on external data sets (Jahandideh et al., 2017) for which population data should ideally be used (Taylor A. A. et al., 2016). Many referral centres develop models based on local datasets (Schuster et al., 2017; van der Burgh et al., 2017; Pfohl et al., 2018), which are more accessible than population-based data. Population-based data are increasingly available thanks to national (Donaghy et al., 2009; Talman et al., 2016) and regional (Rosenbohm et al., 2017) registries and increasingly thanks to international consortia (Turner et al., 2011; Müller et al., 2016; Westeneng et al., 2018).

The direct comparison of model performances in ALS ML studies is challenging as performance metrics, prediction targets, sample sizes and study designs are hugely divergent. There is little evidence that a specific type of input data, clinical features alone or clinical data enriched with other data types, enhances model performance. This is due to the lack of large scale databases which routinely store biological samples and imaging data along with clinical observations. It is likely that the incorporation of genetic, biological, and imaging features, will improve prognostic modeling. Some studies candidly discuss their methodological limitations, and model overfitting is the most often cited shortcoming. Data censoring is often mentioned when using PRO-ACT data and selection bias when relying on clinical trial data. Most studies discuss the issues around feature selection and the importance of limiting feature dimension. Model interpretability concerns are sometimes raised when using NN models (van der Burgh et al., 2017). Westeneng et al. (2018) published their findings according to the methodology introduced by Moons et al. (2015) setting an example of performance reporting. Please refer to Tables 4, 5 for an overview of ML studies in ALS focusing on prognostic projections.

**Table 4:** Research overview: Prognosis with ML models (1/2).

**Key**	**Dataset(s) origin**	**Dataset(s) type**	**Dataset(s) length**	**Scope**	**Biomarker(s) type**	**Pre-processing (if any)**	**Validation (if any)**	**Model(s) tested**	**Performance**	**Framework**
Gomeni and Fava, 2013	PRO-ACT	Clinical trial	338	Progression	Clinical	FS	HOV	non-linear Weibull	AUC:0.96	Classification
Hothorn and Jung, 2014	PRO-ACT	Clinical trial	1822	Progression	Clinical, biological	MVI, VIA	HOV	RF	RMSE:0.52 (ALSFRS rate), PC:40%	Regression
Ko et al., 2014	PRO-ACT	Clinical trial	1822	Progression	Clinical, biological	FS	HOV	RF	Spec:66%, Sens:65%, Acc:66%	Classification
Beaulieu-Jones and Greene, 2016	PRO-ACT	Clinical trial	3398	Outcome	Clinical, biological	MVI	CV	NN, RF, SVM, k-NN, raji DT, NN with RF raji(best)	AUC:0.692	Classification
Taylor A. A. et al., 2016	PRO-ACT, Emery ALS Clinic	Clinical trial, real-life	4372	Progression	Clinical	FS, MVR, VIA	HOV	GLM, RF (best)	*R*^2^:58.2%, MC:0.942, ME:-0.627 (ALSFRS score)	Regression
van der Burgh et al., 2017	University Medical Center Utrecht	Real-life	135	Outcome	Clinical, imaging	SP	HOV	NN	Acc:84.4%	Classification
Huang et al., 2017	PRO-ACT	Clinical trial	6565	Outcome	Clinical, biological	FS, MVR, raji VIA	CV	GP, Lasso, RF (best)	C-ind:0.717	Regression
Jahandideh et al., 2017	PRO-ACT, NEALS	Clinical trial, population	4406	Progression	Clinical, biological	FS, MVI, VIA	CV	RF, XGBoost, GBM (best)	RMSE:0.635 (FVC), *R*^2^:66.9%	Regression
Ong et al., 2017	PRO-ACT	Clinical trial	1568-6355	Progression, outcome	Clinical, biological	MVR, VIA	CV	Boosting	For P: AUC:0.82, rajiAcc:56.5%, rajiSpec:74%, rajiSens:39%, rajiFor O: AUC:0.83, rajiAcc:76.7%, rajiSpec:76.1%, rajiSens:77.3%	Classification

*CV, Cross Validation; HOV, Hold Out Validation; AUC, Area under the ROC Curve; Acc, Accuracy; Sens, Sensitivity; Spec, Specificity; MC, Model Calibration; ME, Mean Error; PC, Pearson's Correlation; DT, Decision Tree; GLM, Generalized Linear Model; k-NN, k-Nearest Neighbors; FS, Feature Selection; MVI, Missing Value Imputation; VIA, Variable Importance Analysis; MVR, Missing Value Removal; P, Progression; O, Outcome; C-ind, Concordance; GP, Gaussian Process; GBM, Gradient Boosting Model; SP, Signal Processing; FVC, Forced Vital Capacity*.

**Table 5:** Research overview: Prognosis with ML models (2/2).

**Key**	**Dataset(s) origin**	**Dataset(s) type**	**Dataset(s) length**	**Scope**	**Biomarker(s) type**	**Pre-processing (if any)**	**Validation (if any)**	**Model(s) tested**	**Performance**	**Framework**
Schuster et al., 2017	Trinity College Dublin	Real-life	69	Outcome	Clinical, imaging	SP, FS	CV	Logistic regression	Spec:83.34%, Sens:75%, Acc:79.19%	Classification
Seibold et al., 2017	PRO-ACT	Clinical trial	2534-3306	Progression, outcome	Clinical, biological	MVR, VIA	None	RF	Treatment effect on rajioutcome and progression	Regression
Bandini et al., 2018	-	Clinical trial	64	Progression	Clinical	SP, FS	CV	k-NN, SVM (best)	Spec:86.1%, Sens:88.8%, Acc:87%	Classification
Pfohl et al., 2018	Emery ALS Clinic	Real-life	801	Outcome	Clinical	MVI, FS, VIA	CV	GLM, raji RF (best)	RMSE:547 raji+/-46 days, raji*R*^2^:52%, rajiAUC:0.85	Regression, Classification
Westeneng et al., 2018	14 European ALS centers	Real-life	11475	Outcome	Clinical	FS, MVI	CV	MRP	Acc:78%, MC:1.01, AUC:0.86	Classification

*CV, Cross Validation; AUC, Area under the ROC Curve; Acc, Accuracy; Sens, Sensitivity; Spec, Specificity; MC, Model Calibration; GLM, Generalized Linear Model; k-NN, k-Nearest Neighbors; MRP, Multivariate Royston-Parmar; FS, Feature Selection; MVI, Missing Value Imputation; VIA, Variable Importance Analysis; MVR, Missing Value Removal;SP, Signal Processing*.

##### Data management approaches

Most studies perform some kind of data pre-processing, such as feature selection (Gomeni and Fava, 2013; Ko et al., 2014; Taylor A. A. et al., 2016; Huang et al., 2017; Jahandideh et al., 2017; Schuster et al., 2017; Bandini et al., 2018; Pfohl et al., 2018; Westeneng et al., 2018), signal processing (Schuster et al., 2017; van der Burgh et al., 2017; Bandini et al., 2018), and address missing data (Hothorn and Jung, 2014; Beaulieu-Jones and Greene, 2016; Taylor A. A. et al., 2016; Huang et al., 2017; Jahandideh et al., 2017; Ong et al., 2017; Seibold et al., 2017; Pfohl et al., 2018; Westeneng et al., 2018). Feature importance analysis prior to model design provides important insights before feature selection (Hothorn and Jung, 2014; Taylor A. A. et al., 2016; Huang et al., 2017; Jahandideh et al., 2017; Ong et al., 2017; Seibold et al., 2017; Pfohl et al., 2018). Feature selection is automated when using RF, NN, or boosting models. Missing data management is crucial when dealing with medical data sets as it has a strong impact on data bias and overall model performance. Huang et al. (2017), Seibold et al. (2017), Taylor A. A. et al. (2016), and Ong et al. (2017) discarded data samples with missing features which can introduce further bias in sparse data situations. Mean imputation, which is a simple imputation method, was performed by Jahandideh et al. (2017) and Hothorn and Jung (2014). Simple imputation methods can increase bias in data as these methods assume missing ‘completely at random’ characteristics which rarely reflect real-life scenarios. Consequently, multiple imputation approaches such as NN approaches (Beaulieu-Jones and Greene, 2016) or MICE (Westeneng et al., 2018) are favored. With few exceptions, Seibold et al. (2017), most studies report their validation framework in detail. Cross-validation schemes are used by some (Beaulieu-Jones and Greene, 2016; Huang et al., 2017; Jahandideh et al., 2017; Ong et al., 2017; Bandini et al., 2018; Pfohl et al., 2018; Westeneng et al., 2018) and hold out validation schemes are implemented by others (Gomeni and Fava, 2013; Hothorn and Jung, 2014; Ko et al., 2014; Taylor A. A. et al., 2016; van der Burgh et al., 2017). Dataset population ranges between 64 and 11 475 samples which explains the considerable methodological differences in pre-processing, data analysis and overall model design. SFR ranges between < 1 (with 135 samples for 2 376 features (van der Burgh et al., 2017)) to close to 1100 (with 6 565 samples for 6 features (Huang et al., 2017)). Small SFRs are mostly due to either data type scarcity (Schuster et al., 2017; van der Burgh et al., 2017; Bandini et al., 2018) or the use of complex models such as NN (Beaulieu-Jones and Greene, 2016). Six studies have used less than nine features for model design (Gomeni and Fava, 2013; Hothorn and Jung, 2014; Ko et al., 2014; Huang et al., 2017; Ong et al., 2017; Westeneng et al., 2018) reaching SFRs over 100 samples per feature.

### 3.4. Advances in Risk Stratification

Accurate patient stratification is not only essential for clinical trial designs but also for individualized patient care (Kiernan, 2018). Current stratification strategies are surprisingly limited and do not utilize patient clustering for pharmaceutical research and medical interventions. Only two drugs have been approved by the FDA to treat ALS to date: Riluzole (Rilutek) and Edavarone (Radicava). While there is some debate if the maximal therapeutic benefit of Riluzole may be in late-stage disease (Dharmadasa et al., 2018; Fang et al., 2018), recent research suggest that Edavarone effect may be superior in the earlier phases of ALS (Goutman, 2017; Kiernan, 2018). It is also noteworthy, that past clinical trials were primarily based on heterogeneous ALS populations. The inconclusive findings of admixed cohorts may not apply to specific patient subgroups (Bozik et al., 2014) or presymptomatic cohorts. Rigorous patient stratification would have an important role in addressing these shortcomings. Unsupervised learning methods, such as the one carried out by Beaulieu-Jones and Greene (2016) using denoised autoencoder and t-distributed Stochastic Neighbor Embedding (t-SNE), provide novel means of monitoring patients. However, as for most unsupervised learning methods, selecting the appropriate number of patient clusters requires extensive empirical testing.

#### 3.4.1. Overview of Stratification Initiatives

Patient stratification in ALS is often explored from a prognostic perspective (Visser et al., 2007; Gomeni and Fava, 2013; Ko et al., 2014; Elamin et al., 2015; Marin et al., 2015; Beaulieu-Jones and Greene, 2016; Ong et al., 2017; van der Burgh et al., 2017; Pfohl et al., 2018; Westeneng et al., 2018) approaching it as a classification problem and patient categories are defined to build the model. Balendra et al. (2014a) analyzed progression patterns using the King's staging system. Clinical stages are potential input variables for stratification, and therapeutic intervention can be tested based on disease subgroups or disease stage.

Patient stratification was performed based on clinical observations alone in seven recent studies (Visser et al., 2007; Balendra et al., 2014a; Ko et al., 2014; Elamin et al., 2015; Burke et al., 2017; van der Burgh et al., 2017; Pfohl et al., 2018). Variables, such as limb involvement (Visser et al., 2007), disease-stage (Balendra et al., 2014a), ALSFRS-r decline (Ko et al., 2014), executive dysfunction (Elamin et al., 2015), behavioral impairment (Burke et al., 2017), and survival (van der Burgh et al., 2017; Pfohl et al., 2018) have been used for patient stratification. Other studies relied on unsupervised techniques to identify patient subgroups. These methods either used model estimation (Gomeni and Fava, 2013; Westeneng et al., 2018), K-means (Ong et al., 2017), a tree-growing algorithm called Recursive Partitioning and Amalgation (Marin et al., 2015) or NNs with a denoising autoencoder (Beaulieu-Jones and Greene, 2016). Clustering was performed either based on clinical features alone (Gomeni and Fava, 2013; Marin et al., 2015; Westeneng et al., 2018) or based on clinical features and biological data (Beaulieu-Jones and Greene, 2016; Ong et al., 2017).

Contrary to supervised learning problems, unsupervised learning methods do not have clear and easily presentable performance metrics. Possible options include the description of inter- and intra-patient subgroup distances and outlier distribution. The optimal number of models (equivalent to cluster number) can be identified using an iterative procedure for studies based on model estimation (Gomeni and Fava, 2013; Westeneng et al., 2018).

##### Clustering methods

Patient clustering was performed on various datasets in ALS; clinical trial data (Gomeni and Fava, 2013; Balendra et al., 2014a; Ko et al., 2014; Ong et al., 2017), “real-life data” (Visser et al., 2007; van der Burgh et al., 2017; Pfohl et al., 2018; Westeneng et al., 2018) and population data (Elamin et al., 2015; Marin et al., 2015; Burke et al., 2017). The term “real-life” data is used to samples which derive from local recruitment, typically single-center non-pharmacological studies, where data are acquired prospectively but do not represent entire populations. Access to large patient databases with limited missing data is fundamental to the development of accurate stratification schemes. Recent initiatives such as the Prize4Life challenge (Küffner et al., 2014), the PRO-ACT database and Euro-MOTOR consortium (Rooney et al., 2017; Visser et al., 2018) have proven invaluable resources for research and should be continued and expanded. PRO-ACT's main limitation with regards to patient stratification is its inclusion bias. Working with population data leads to more representative results as clinical trial datasets tend to be associated with considerable bias. The identification of specific patient subgroups is most accurate when the data truly represents an entire patient population.

The maximum number of clusters does not typically exceed five in ALS research; Gomeni and Fava (2013), Ko et al. (2014), Beaulieu-Jones and Greene (2016), Ong et al. (2017), and Pfohl et al. (2018) work with only two patient subgroups, Visser et al. (2007), Elamin et al. (2015), van der Burgh et al. (2017), and Burke et al. (2017) with three patient subgroups, Marin et al. (2015) with four patient subgroups and Balendra et al. (2014a); Westeneng et al. (2018) with five patient subgroups. Depending on the available data, feature type, and data source working with a limited number of clusters may be desirable. This can be particularly challenging in ALS, where a number of phenotypes contribute to clinical heterogeneity. Identifying the correct number of clusters is a common problem in unsupervised learning which can only be solved with *ad-hoc* analyses. Please refer to Tables 6, 7 for an overview of studies focusing on risk stratification in ALS.

**Table 6:** Research overview: Patient stratification (1/2).

**Key**	**Dataset(s) origin**	**Dataset(s) type**	**Dataset(s) length**	**Scope**	**Approach**	**Clustering feature(s)**	**Number of clusters found**
Visser et al., 2007	Dutch university hospitals	Real-life	37	Progression	Clinical observations	Limb involvement	3
Gomeni and Fava, 2013	ProACT	Clinical trial	338	Progression	Unsupervised (non-linear Weibull model rajiestimation)	Clinical features	2
Balendra et al., 2014a	LiCALS, Mito Target	Clinical trial	725	Progression	Clinical observations	Clinical stages	5
Ko et al., 2014	ProAct	Clinical trial	1822	Progression	Clinical observations	ALSFRS decline rate	2
Elamin et al., 2015	Irish ALS registry, Italy (Piemonte Region)	Population	326	Outcome	Clinical observations	Score based on onset type, ALSFRS rate an executive disfunction	3
Marin et al., 2015	FRALim register	Population	322	Outcome	Unsupervised (RECPAM)	Clinical features	4

*RECPAM, Recursive Partitioning and Amalgation*.

**Table 7:** Research overview: Patient stratification (2/2).

**Key**	**Dataset(s) origin**	**Dataset(s) type**	**Dataset(s) length**	**Scope**	**Approach**	**Clustering feature(s)**	**Number of clusters found**
Beaulieu-Jones and Greene, 2016	ProAct	Clinical trial	3398	Outcome	Unsupervised learning (DA)	Clinical and biological features	2
van der Burgh et al., 2017	University Medical Center rajiUtrecht	Real-life	135	Outcome	Clinical observations	Survival time based on Elamin2015 categories	3
Burke et al., 2017	Irish ALS Register	Population	383	Progression	Clinical observations	Behavioral rajiimpairment based on BBI score	3
Ong et al., 2017	ProAct	Clinical trial	1568-6355	Progression, rajioutcome	Unsupervised raji(PAM and K-Means)	Clinical and biological features	2x2
Pfohl et al., 2018	Emery ALS Clinic	Real-life	801	Outcome	Clinical observations	Survival time raji(empirical)	2
Westeneng et al., 2018	14 European ALS centers	Real-life	11475	Outcome	Unsupervised raji(RP model estimation)	Clinical features	5

*DA, Denoising Autoencoders; PAM, Partitioning Around Medoids; RP, Royston-Parmar; BBI, Beaumont Behavioral Inventory*.

ALS studies approach patient stratification in strikingly different ways. Visser et al. (2007) proposed an innovative PMA strategy which is based on limb involvement and focuses on symmetrical vs. asymmetrical limb weaknesses. Current ALS phenotyping already considers aspects of limb involvement, but this could be extended to adopt more detailed characterization. Gomeni and Fava (2013) divided patients into slow- and fast-progressing groups based on non-linear Weibull model estimation, which can account for linear, sigmoid or exponential evolutions. Two clusters were retained based on model fitting, as three-cluster attempts proved less conclusive. Balendra et al. (2014a) explored King's stages (Roche et al., 2012) on LiCALS and Mito Target data and demonstrated a viable alternative to ALSFRS-r and traditional patient stratification strategies. Clinical staging is thought to represent pathological stages better than ALSFRS-r. Alternative clinical staging systems, such as MiToS (Chiò et al., 2013a) or Fine'Till 9 (Thakore et al., 2018) could be tested further to assess if they are more sensitive in the earlier or later stages of the disease. Ko et al. (2014) performed an interesting patient classification study based on ALSFRS-r decline but choice of threshold, 0.6 ALSFRS-r point / month was not expounded. Elamin et al. (2015) divided patients into three risk groups based on a scoring system, which was based on site of onset, ALSFRS-r, and executive dysfunction. Marin et al. (2015) identified four groups using an unsupervised ML technique: Recursive partitioning and amalgamation. Membership rules were derived from analyzing ALSFRS-r decline and El Escorial criteria. Beaulieu-Jones and Greene (2016) investigated PRO-ACT survival data using denoising autoencoders, a deep learning model, and used the visualization algorithm t-SNE to visualize how the NN model had divided the subjects according to short vs. long survival. These results are particularly promising as NN models can work well without extensive feature selection. van der Burgh et al. (2017) segregated patients into three classes based on survival times defined by Elamin et al. (2015). Burke et al. (2017) proposed three subgroups for clustering based on executive dysfunction (“non-significant,” “mild,” and “severe symptoms”) using the Beaumont Behavioral Inventory (Elamin et al., 2016), a questionnaire on patient behavior completed by the patient and caregivers. Ong et al. (2017) used unsupervised ML techniques Partitioning Around Medoids and K-Means to identify patient clusters for disease progression and survival. Partitioning Around Medoids and K-Means differ on cluster computing as the former computes the medoid (data point whose average dissimilarity with the other data points is minimal) while the latter computes the average value. Two clusters were optimally suited for both algorithms. Pfohl et al. (2018) used empirically defined survival times based on clinician experience. Westeneng et al. (2018) identified five patient groups after Royston-Parmar model analysis and estimation. Differing patient stratification strategies can be successfully combined as demonstrated by Burke et al. (2017) who analyzed cognitive impairment stratification with regards to King's clinical staging system.

## 4. Discussion

### 4.1. Summary of Main Findings

#### 4.1.1. Diagnosis

ML models have been increasingly explored in diagnostic applications in ALS. These models have the potential to supersede the current gold standard diagnostic approach which is based on clinical evaluation and uses the El Escorial criteria. The El Escorial criteria is thought to suffer from low specificity (Goutman, 2017). Recent ML models in ALS have reached comparable sensitivity and specificity values to the El Escorial criteria. The main barriers to model performance stem from limited data availability for training and poor sample to feature ratios. Future strategies should centre on models using multimodal data, and models which discriminate phenotypes within the ALS spectrum and distinguish ALS from disease-controls. Optimally, these models should be developed to enable an early, definite, and observer independent diagnosis of ALS.

#### 4.1.2. Prognosis

The development of accurate prognostic models attracts considerable interest, and is fuelled by initiatives like the challenge launched by Prize4Life (Küffner et al., 2014). Prognostic model performance depends heavily on each feature's relevance to disease propagation. Current models rely primarily on clinical findings and laboratory tests which might not be sufficient to predict disease evolution. Despite these challenges, recent models have provided a reasonable gross estimate of death risk (Ong et al., 2017), survival (Schuster et al., 2017; van der Burgh et al., 2017; Westeneng et al., 2018) and progression rates (Ong et al., 2017). The most important constraints of prognostic modeling stem from significant data bias, limited data availability, poor missing data management, and limited sample to feature ratios. Performance reporting should be standardized for model comparisons, reproducibility, and benchmark development. Future studies should include multimodal data, multiple timepoints, include ALS patients with comorbid FTD and appraise disease progression in terms of clinical stages instead of solely relying on ALSFRS-r. Effective prognostic modeling should also account for disease heterogeneity to provide patients and clinicians with accurate prognostic insights across multiple phenotypes.

#### 4.1.3. Risk Stratification

Novel computerized risk stratification initiatives are urgently required in ALS, as this aspect of ALS research has been relatively ignored to date. Existing studies tend to stratify patients according to rather basic categorization rules, limiting their analyses to a restricted number of clusters and focusing mostly on clinical features. Future research should focus on working with multimodal and longitudinal datasets and analyzing model-derived clustering with commonly used ALS phenotypes. Optimized patient stratification schemes will undoubtedly improve clinical trial design and has the potential to identify clinically relevant ALS subtypes.

## 5. Conclusions

ML models have enormous academic and clinical potential in ALS. With the increasing availability of large datasets, multicentre initiatives, high-performance computer platforms, open-source analysis suites, the insights provided by flexible ML models are likely to supersede those gained from conventional statistical approaches. The choice of the ML model need to be carefully tailored to a proposed application based on the characteristics of the available data and the flexibility, assumption and limitation profile of the candidate model. While ALS research to date has overwhelmingly relied on conventional ML approaches, emerging models and neural network architectures have considerable potential to advance the field. Novel models such as “black box” methods however may suffer from similar pitfalls than established algorithms. The meticulous evaluation of data characteristics, appraisal of data bias, missing data, sample to feature ratio is indispensable irrespective of the choice of ML model. Novel models may have outperformed traditional approaches, but data constraints and limitations are often overlooked. Model overfitting is the most commonly encountered shortcoming of recent studies which limits the generalizability of a proposed model. Transparent performance assessment using standardized metrics, robust missing data management and adherence to reporting guidelines are key requirements for future machine learning studies in ALS. Despite the drawbacks of current models and the methodological limitations of recent studies, the momentous advances in the field suggest that ML models will play a pivotal role in ALS research, drug discovery, and individualized patient care.

## Author Contributions

VG contributed to the design of the study, analyzed the data, and wrote the first draft of the manuscript. VG, GL, PB, FD, J-FP-P, and P-FP contributed to the revision of the manuscript. VG, GL, PB, FD, J-FP-P, P-FP, and GQ read and approved the final version.

### Conflict of Interest Statement

The authors declare that the research was conducted in the absence of any commercial or financial relationships that could be construed as a potential conflict of interest.

## Glossary

ALS: Amyotrophic Lateral Sclerosis
ALSbi: Behaviorally impaired ALS
ALSFRS: ALS Functional Rating Scale
ALSbi: behaviorally impaired ALS
ALSnci: ALS with no cognitive impairment
ALSci: ALS with cognitive impairment
ALSexec: ALS with executive dysfunction
AUC: Area Under the ROC Curve
AD: Axial Diffusivity
CNN: Convolutional Neural Network
CSF: Cerebrospinal fluid
CST: Corticospinal
DeepCNF: Deep Convolutional Neural Fields
DTI: Diffusion Tensor Imaging
FA: Fractional Anisotropy
FTD: Frontotemporal Dementia
GMM: Gaussian Mixture Model
KD: Kennedy's disease
k-NN: k-Nearest Neighbors
LMN: Lower Motor Neurons
MD: Mean Diffusivity
ML: Machine Learning
MND: Motor Neuron Disease
NN: Neural Network
PBP: Progressive Bulbar Palsy
PCA: Principal Component Analysis
PD: Parkinson's Disease
PLS: Primary Lateral Sclerosis
PMA: Progressive Muscular Atrophy
PRO-ACT: Pooled Resource Open-Access ALS Clinical Trials
RBP: RNA-Binding Protein
RD: Radial Diffusivity
RF: Random Forest
RMSE: Root Mean Squared Error
RNN: Recurrent Neural Network
ROC: Receiver Operating Curve
SFR: Sample to Feature Ratio
SMA: Spinal Muscular Atrophy
SVM: Support Vector Machine
t-SNE: t-distributed Stochastic Neighbor Embedding
UMN: Upper Motor Neurons
